# Porcine reproductive and respiratory syndrome virus non-structural protein 4 cleaves guanylate-binding protein 1 via its cysteine proteinase activity to antagonize GBP1 antiviral effect

**DOI:** 10.1186/s13567-022-01071-8

**Published:** 2022-07-08

**Authors:** Hong Duan, Haoxin Dong, Shuya Wu, Jiahui Ren, Mingfang Zhang, Chuangwei Chen, Yongkun Du, Gaiping Zhang, Angke Zhang

**Affiliations:** 1 College of Veterinary Medicine, Henan Agricultural University, Zhengzhou, 450046 Henan China; 2 International Joint Research Center of National Animal Immunology, Henan Agricultural University, Zhengzhou, 450046 Henan China

**Keywords:** PRRSV, GBP1, GTPase activity, nsp4 protein, cleavage effect

## Abstract

Porcine reproductive and respiratory syndrome (PRRS) is a highly infectious disease caused by PRRS virus (PRRSV) that causes great economic losses to the swine industry worldwide. PRRSV has been recognized to modulate the host antiviral interferon (IFN) response and downstream interferon-stimulated gene expression to intercept the antiviral effect of host cells. Guanylate-binding proteins (GBPs) are IFN-inducible GTPases that exert broad antiviral activity against several DNA and RNA viruses, of which GBP1 is considered to play a pivotal role. However, the role of GBP1 in PRRSV replication remains unknown. The present study showed that overexpression of GBP1 notably inhibited PRRSV infection, while the knockdown of endogenous GBP1 promoted PRRSV infection. The K51 and R48 residues of GBP1 were essential for the suppression of PRRSV replication. Furthermore, GBP1 abrogated PRRSV replication by disrupting normal fibrous actin structures, which was indispensable for effective PRRSV replication. By using a co-immunoprecipitation assay, we found that GBP1 interacted with the non-structural protein 4 (nsp4) protein of PRRSV, and this interaction was mapped to the N-terminal globular GTPase domain of GBP1 and amino acids 1–69 of nsp4. PRRSV infection significantly downregulated GBP1 protein expression in Marc-145 cells, and nsp4, a 3C-like serine proteinase, was responsible for GBP1 cleavage, and the cleaved site was located at glutamic acid 338 of GBP1. Additionally, the anti-PRRSV activity of GBP1 was antagonized by nsp4. Taken together, these findings expand our understanding of the sophisticated interaction between PRRSV and host cells, PRRSV pathogenesis and its mechanisms of evading the host immune response.

## Introduction

Porcine reproductive and respiratory syndrome (PRRS) is one of the most important infectious diseases in the swine industry worldwide [1]. The causative pathogen is PRRS virus (PRRSV), which belongs to the genus *Arterivirus* and is a member of the family Arteriviridae and order Nidovirales, is an enveloped, single-stranded positive RNA virus [2]. The PRRSV genome is ~15 kb in length, with at least 10 open reading frames (ORFs) that encode two polyprotein precursors (pp1a and pp1ab) and eight structural proteins (GP2, GP3, GP4, GP5, ORF5a, M, ORF7 and E) [3]. Once the replication process is initiated inside the host cell, pp1a and pp1ab, encoded by the 5′ two-thirds of the viral genome, are proteolytically processed into at least 14 functional non-structural proteins (nsp, nsp1 to nsp12, with nsp1 autocleaved into nsp1α/nsp1β and nsp7 autocleaved into nsp7α/nsp7β) [4]. Nsp4, a 3C-like serine protease (3CLSP), plays a significant role in the processing of pp1a and pp1ab to release nsp3 to nsp12 from pp1a and pp1ab [5]. The 3′ end of the viral genome encodes envelope proteins (GP2, GP3, GP4, GP5, ORF5a, E and M) and also nucleocapsid (N) protein that encapsulates the genomic RNA [6]. Those proteins are responsible for PRRSV genome replication and transcription, and some of these proteins participate in modulating host immune responses.

To replicate and spread successfully, viruses have evolved various strategies to escape host defense systems. PRRSV has been found to suppress interferon (IFN) production activated by double-stranded RNA [7, 8]. Conversely, certain PRRSV proteins, such as nsp1β and nsp11, have been identified and characterized as IFN antagonists via lysosomal degradation of cholesterol-25-hydroxylase [9]. Furthermore, both PRRSV nsp2 and nsp11 play important roles in suppressing host innate immune responses [10, 11]. PRRSV nsp4 was reported to inhibit IFN-I production via destruction of the IFN signaling pathway, which was dependent on its 3CLSP activity [12–14]. These studies highlight the important role of PRRSV nsps in the process of innate immune evasion by viruses. As key components of the innate immune system, IFN-I provides critical defenses against viral pathogens [15]. Rapid and robust induction of IFN-I is a key event during host antiviral responses. The binding of IFN-I to the IFN-α/β receptor initiates a signaling cascade that leads to the induction of  > 300 interferon-stimulated genes (ISGs). ISGs directly target multiple stages of the viral life cycle to resist viral infection, and thus help host cells establish an effective antiviral state. Some of these ISGs, such as ISG15, Mx1/2, IFITM3 and OAS/RNaseL, have been shown to play important roles in the host defense against PRRSV infection [16–19]. Zinc-finger antiviral protein (ZAP) is also an ISG, and PRRSV nsp4 antagonizes the antiviral activity of ZAP by cleaving it through its 3CLSP activity [20]. mRNA-decapping enzyme 1a (DCP1a) was reported to be an ISG that could obstruct PRRSV replication, while nsp4 could antagonize its antiviral effect by cleaving DCP1a [21]. However, the specific functions of many of the ISGs are either unknown or only poorly characterized.

Guanylate-binding proteins (GBPs) belong to a family of large GTPases that includes dynamins and Mx proteins [22]. To date, seven human GBPs (hGBP1–hGBP7) have been identified, all of which are assumed to act as GTPases hydrolyzing guanosine triphosphate (GTP) to both guanosine diphosphate and guanosine monophosphate, and were originally identified as the most abundant proteins induced by IFN treatment in human fibroblasts [23, 24]. GBPs are involved in important cellular processes, including signal transduction, translation, vesicle trafficking and exocytosis [25]. An increasing number of studies have shown that GBPs are likely to be the most unexplainable of all IFN-inducible GTPases. They were initially described as cellular factors providing resistance against bacterial and protozoan pathogens [26], and were subsequently also shown to be important components of the immune defense against viruses. Among the GBP family, GBP1 is considered to be an ISG that is composed of an N-terminal globular GTP-binding domain and a C-terminal helical domain, which contains seven helices. It is a large self-activating GTPase, and the dimerization is necessary for GTP-hydrolyzing activity; additionally, studies have reported that the GTPase activity of GBP1 is responsible for effective antiviral activity [27–29]. For example, previous studies indicated that GBP1 can provide defense against various RNA viruses, such as hepatitis C virus (HCV), dengue virus, vesicular stomatitis virus, classical swine fever virus and influenza A virus (IAV) [28–32]. Since viral diseases have caused substantial economic harm to the porcine industry, noteworthy candidate molecules to act in the host defense against PRRSV infection have evoked considerable interest, and the roles of the GBP family, to a large extent, are still yet to be fully elucidated. So far, only GBP1 and GBP2 genes have been identified in porcine animals. Porcine GBP1 gene has been shown to be associated with resistance of pigs to PRRSV infection according to cell model to animal models analysis using next-generation sequencing [33, 34]. Although numerous studies have characterized the restriction of viruses by the GBP1 protein, little is known about the antiviral activity and the underlying mechanism of GBP1 against PRRSV.

In the present study, the effect of GBP1 on PRRSV infection and the underlying mechanism was investigated. The results showed that overexpression of GBP1 suppressed PRRSV replication, while knockdown of endogenous GBP1 expression promoted PRRSV replication. Mechanical studies showed that GBP1 suppressed PRRSV replication via disruption of actin morphological integrity, which was proven to be essential for PRRSV replication. Additionally, GBP1 was found to interact with PRRSV nsp4, and this interaction was primarily located at the N terminal of GBP1. Furthermore, the GTPase activity of GBP1 was shown to be necessary for GBP1-nsp4 interaction. Further studies suggested that nsp4 antagonized the antiviral activity of GBP1 by cleaving it at the E338 site via its 3CLSP activity. Thus, this study revealed a novel mechanism underlying the evasion of the host antiviral immune system by PRRSV.

## Materials and methods

### Cells, virus, chemical reagents and antibodies

African green monkey kidney cells (Marc-145, the only cell line that are fully permissive to PRRSV replication in vitro) [35] and human embryonic kidney (HEK293T) cells were cultured in Dulbecco’s modified Eagle’s medium (DMEM; Lonza Group, Ltd., Basel, Switzerland) supplemented with 10% (vol/vol) fetal bovine serum (FBS; Hyclone, Danaher Corporation, Washington, USA), 100 μg/mL streptomycin and 100 IU/mL penicillin. Porcine alveolar macrophages (PAMs) were obtained from healthy 6-week-old crossbred weaned (Landrace Yorkshire) PRRSV-negative pigs by using a lung lavage technique as previously described [36] and maintained in RPMI-1640 medium (Gibco, Thermo Fisher Scientific, Inc., Waltham, MA, USA) supplemented with 10% FBS and penicillin–streptomycin. All cells were cultured and maintained at 37 °C with 5% CO_2_. All animal experiments were performed in strict accordance with the guidelines of the Institutional Animal Care and Use Committee and approved by the Animal Care and Use Committee of Henan Agricultural University (DWLL3086427.13).

The highly pathogenic PRRSV SD16 strain (GenBank Accession No. JX087437.1) was used for all experiments and is the strain represented by “PRRSV” in this article unless otherwise specified. Enhanced green fluorescent protein (EGFP)-PRRSV based on the genetic background of the SD16 strain that expressed EGFP as an additional ORF (rHP-PRRSV/SD16/EGFP) was provided by professor En-min Zhou of Northwest A&F University [37]. The virus was propagated, titrated in Marc-145 cells and stored at −80 °C.

The proteasome inhibitor MG-132 and lysosome inhibitor 3-Methyladenine (3-MA) were purchased from MedChemExpress (New Jersey, USA). A pan-caspase inhibitor Z-VAD-FMK was purchased from Beyotime Institute of Biotechnology (Shanghai, China). Microfilament disruptor cytochalasin D, which depolymerizes actin, was purchased from Sigma-Aldrich (Merck KGaA, Darmstadt, Germany).

Monoclonal antibody (mAb) against PRRSV N protein was produced in our lab. Anti-flag (Catalog No. AF0036) and -HA tag (Catalog No. AF5057) antibodies were both obtained from Beyotime Institute of Biotechnology. Anti-α-tubulin mAb was purchased from Sigma-Aldrich (Catalog No. T6199; Merck KGaA). HRP-conjugated goat anti-mouse (Catalog No. 115-035-003) or -rabbit IgG (Catalog No. 111-035-045) secondary antibodies were purchased from Jackson ImmunoResearch Laboratories, Inc (West Grove, PA, USA). Alexa Fluor 488-conjugated goat anti-rabbit IgG (H&L) (Catalog No. ab150077) and Alexa Fluor 594-conjugated goat anti-mouse IgG (H&L) (Catalog No. ab150116) were obtained from Abcam (Cambridge, UK). Purified recombinant human IFN-γ (100 μg/mL) was provided by Beyotime Institute of Biotechnology.

### Generation of Marc-145 cells stably expressing porcine GBP1

cDNA encoding porcine GBP1 was amplified using reverse transcription-PCR (RT-PCR) from total RNAs extracted from PAMs and cloned into pTRIP-puro lentiviral expression vector to construct the recombinant plasmid pTRIP-GBP1-flag. pTRIP-GBP1-flag (0.9 μg) vector was co-transfected into HEK293T cells with packaging vectors psPAX (1.8 μg) and pMDG.2 (1.0 μg) to produce pseudotyped lentiviral vectors using X-tremeGENE HP DNA transfection reagent (Roche Diagnostics, Mannheim, Germany). At 60 h post-transfection (hpt), cell supernatants containing the pseudotyped lentiviral vectors were collected and centrifuged at 12 000 × *g* at 4 °C for 10 min to wipe off cell debris. A blank pTRIP-puro vector was used as a control. To generate Marc-145 cells stably expressing GBP1, cell supernatants containing the pseudotyped virus were co-incubated with Marc-145 cells for 24 h before the old medium was replaced with 10% FBS + DMEM containing 8 μg/mL puromycin (Sigma-Aldrich; Merck KGaA). The old culture medium was changed every 48 h. Marc-145 cell lines with different mutant versions of GBP1 (K51A, R48A, ΔCAAX) were constructed by cloning mutant genes into pTRIP-puro using overlap PCR with mutant primers, and all cell lines were selected using puromycin screening and identified by Western blotting.

### RNA isolation and RT-quantitative PCR (RT-qPCR)

Total RNA was extracted from cells using TRIzol^®^ reagent (Invitrogen; Thermo Fisher Scientific, Inc.) and reverse transcribed using a PrimeScript RT reagent kit (Takara Biotechnology Co., Ltd., Dalian, China) according to the manufacturer’s protocols. qPCR was performed using the StepOne Plus real-time PCR system (Applied Biosystems; Thermo Fisher Scientific, Inc.) and FastStart Universal SYBR green master (Roche Diagnostics). The following thermocycling conditions were used for qPCR: initial denaturation at 95 °C for 10 min; 40 cycles at 95 °C for 20 s, 55 °C for 30 s and 72 °C for 20 s. The porcine β-actin gene was used as a reference gene to normalize gene expression. The relative expression of the target genes were calculated using the 2^−ΔΔCq^ method. All primers used for qPCR were designed using primer premier 5.0 (Premier Biosoft International, San Francisco, USA) and shown in Table 1.

**Table 1:** **Primary primers used in this study.**

Primer name	Sequence (5′–3′)
Nsp4-F	CGGGGTACCATGGGTGCTTTCAGAACTCAAAAGC
Nsp4-R	CCGCTCGAGTTATTCCAGTTCGGGTTTGGCAGCAAGC
pGBP1-F	CCCTCGAGATGGATTACAAGGATGACGACGATAAGATGGCCTCAAAGGTGCACAT
pGBP1-R	CGGGATCCTTAGCTCAGGAAACATTCTT
pGBP1_1-290_-F	CCCTCGAGATGGATTACAAGGATGACGACGATAAGATGGCCTCAAAGG TGCACAT
pGBP1_1-290_-R	CGGGATCCTTACAGACGGGGCCCGGTGACC
pGBP1_288-591_-F	CCCTCGAGATGGATTACAAGGATGACGACGATAAGCCCCGTCTGGAGACCCTGGTGC
pGBP1_288–591_-R	CGGGATCCTTAGCTCAGGAAACATTCTT
pGBP1-K51A-F1	CCCTCGAGATGGATTACAAGGATGACGACGATAAGATGGCCTCAAAGG TGCACAT
pGBP1-K51A-R1	CATCAGGTAGGATGCGCCTGTGCGGTAC
pGBP1-K51A-F2	GTACCGCACAGGCGCATCCTACCTGATGAAC
pGBP1-K51A-R2	CGGGATCCTTAGCTCAGGAAACATTCTT
pGBP1-R48A-F1	CCCTCGAGATGGATTACAAGGATGACGACGATAAGATGGCCTCAAAGGTGCACAT
pGBP1-R48A-R1	GATTTGCCTGTTGCGTACAGGCCCAC
pGBP1-R48A-F2	GTGGGCCTGTACGCAACAGGCAAATC
pGBP1-R48A-R2	CGGGATCCTTAGCTCAGGAAACATTCTT
pGBP1-ΔCAAX-F	CCCTCGAGATGGATTACAAGGATGACGACGATAAGATGGCCTCAAAGG TGCACAT
pGBP1-ΔCAAX-R	CGGGATCCTTACTTTGATTTTTTCATCTTCTTC
ORF7-qF	AGATCATCATCGCCCAACAAAAC
ORF7-qR	GACACAATTGCCGCTCACTA
pGBP1-qF	AAGTCAGCCGAGTCCTCAATCC
pGBP1-qR	GCAGCACCTTCGTCTACAACAG
β-actin-qF	TCCCTGGAGAAGAGCTACGA
β-actin-qR	AGCACTGTGTTGGCGTACAG

For PRRSV RNA detection in supernatants, a plasmid containing PRRSV ORF7 sequence was used to generate a standard curve. The standard curve was plotted from the results of parallel PCRs performed on serial dilutions of standard DNA. Absolute quantities of supernatant RNA were calculated by normalization to the standard curve.

### Immunofluorescence assay (IFA)

For the IFA, Marc-145-Vector or Marc-145-GBP1-flag cells were infected with 0.1 MOI of PRRSV and incubated at 37 °C for 1 h. After washing with PBS, cells were further cultured with 3% FBS + DMEM. At 24 h post-infection (hpi), cells were fixed for 10 min with 4% buffered paraformaldehyde and permeabilized in 0.1% Triton X-100 for 3 min at RT. After washing with PBS three times, cells were incubated with anti-flag pAb (1:300) and anti-N protein mAb (1:300) at RT for 1 h followed by washing with PBS three times. Then, cells were stained with Alexa Fluor 488-conjugated goat anti-rabbit IgG (H&L) (1:500) and Alexa Fluor 594-conjugated goat anti-mouse IgG (H&L) (1:500) for 1 h at RT in the dark. Nuclei were counterstained with DAPI (1:5000). Immunofluorescence was observed using a fluorescence microscope (Olympus Corporation, Tokyo, Japan). Mock-infected cells were used as controls to establish background staining levels.

### Confocal analysis

To analyze the effect of GBP1 and its mutants on intracellular actin morphology, Marc-145-Vector or Marc-145-GBP1-flag cells on coverslips were fixed, permeabilized and blocked as described above, followed by staining with rabbit anti-flag pAb (1:300) and Alexa Fluor 488-conjugated goat anti-rabbit IgG (H&L), actin was stained with phalloidin-Alexa Fluor 594 actin tracker (1:200; Beyotime Institute of Biotechnology). After washing with PBS, the coverslips were mounted on glass slides with DAPI counterstain (Sigma-Aldrich; Merck KGaA) and imaged using a Zeiss LSM510 laser scanning inverted confocal microscope with a Zeiss 63x/1.4NA oil lens objective (Zeiss AG, Oberkochen, Germany).

To analyze the subcellular localization of GBP1 and nsp4, HEK293T cells were seeded on coverslips in 24-well plates at a density of 1 × 10^5^ cells/well 24 h prior to co-transfection with pTRIP-GBP1-flag (500 ng/well) and pCAGGS-nsp4-HA (500 ng/well) for 48 h. Transfected cells were fixed with 4% paraformaldehyde for 30 min at RT, followed by permeabilization with 0.1% Triton X-100 for 3 min and blocking with 1% bovine serum albumin for 30 min, and then incubation with rabbit anti-flag pAb (1:300) or mouse anti-HA mAb (1:500) for 2 h at 37 °C. After washing with PBS, the cells were incubated with Alexa Fluor 488-conjugated goat anti-rabbit IgG (H&L) and Alexa Fluor 594-conjugated goat anti-mouse IgG (H&L) (1:500) for 1 h at RT in the dark. To determine whether PRRSV nsp4 interacts with GBP1 during viral replication, Marc-145-GBP1-flag cells were seeded on coverslips in 24-well plates at a density of 1 × 10^5^ cells/well 24 h prior to infecting or mock infecting with 0.1 MOI of PRRSV. At 24 hpi, cells were fixed and stained with the mouse anti-nsp4 pAb (1:300) and rabbit anti-flag pAb (1:300), followed by incubation with the corresponding fluorescent secondary antibody.

### PRRSV intercellular spread assay

A cell co-culture system was utilized to assess the effect of GBP1 on PRRSV intercellular spread. Marc-145 cells were plated in 6-well plate, when cells reached 80% confluence, cells were inoculated with 0.1 MOI of GFP-PRRSV, when they reached ~80% GFP-PRRSV-positive, cells were digested with 0.25% trypsin to obtain a single cell suspension and then the GFP-PRRSV-positive cells were separated using a FACSAria cell sorter and were used as the donor cells. Then the Marc-145-Vector-tomato [red fluorescent protein (RFP) positive] cells and Marc-145-GBP1-tomato (RFP positive) cells were digested with trypsin to obtain a single cell suspension and both were used as the target cells. For the virus cell-to-cell transmission assay, after counting by using trypan blue staining, 4 × 10^5^ GFP-PRRSV positive Marc-145 cells were mixed with an equal number Marc-145-Vector-tomato cells or Marc-145-GBP1-tomato cells (~1:1 donor:target), respectively, and co-cultured at 37 °C for 2 h in 6-well plates. After digesting with 0.25% trypsin to make cells sufficiently dispersed into individual cells,  ~4 × 10^5^ target cells (red) were separated from donors by flow cytometry sorting and were plated in a 6-well plate and culturing was continued for 24 h, following which the cells were observed and photographed under a fluorescence microscope directly. The GFP + RFP + double positive Marc-145 cells can be used for evaluation of viral cell-to-cell transmission.

### Small interfering (si)RNA transfection

To knock down endogenous expression of GBP1 in PRRSV natural permissive cell-PAMs, siRNA targeting two different sites of GBP1 (siGBP1, 5′-CGGCUGACUUUGUGAGCUUTT3′ and 5′-CAGAGAGAAUCAGGGCAAAT T-3′) and universal negative control siRNA (siNC) were transfected into PAMs using X-tremeGENE siRNA Transfection Reagent (Roche Diagnostics) at a final concentration of 100 nM. At 36 hpt, cells were harvested to determine the mRNA and protein levels of GBP1 by qPCR and Western blotting, respectively. To evaluate the effect of GBP1 knockdown on PRRSV replication in PAMs, at 12 h post-siGBP1 and siNC transfection, PAMs were infected with 0.01 MOI of PRRSV. Cells were collected at 36 hpi to determine the expression of PRRSV ORF7 using qPCR and the expression of N protein via Western blotting.

For siRNA and IFN-γ co-incubation, Marc-145 cells were pre-treated with or without 50 U/mL IFN-γ for 12 h, followed by transfection with or without siGBP1 or siNC at a final concentration of 100 nM for another 12 h before cells were infected with 0.1 MOI PRRSV. At 36 hpi, cells were harvested for PRRSV N protein and GBP1 protein expression detection via Western blotting. α-tubulin was detected simultaneously as the internal control.

### Construction of plasmids and transfection of cells

The GBP1 mutants GBP1-K51A, GBP1-R48A and GBP1-ΔCAAX were constructed by overlap PCR. To facilitate the detection of GBP1 expression or its corresponding truncation mutants, an N-terminal flag-tag was introduced via the forward PCR primer. GBP1 glutamic acid site mutants were cloned into the pTRIP-puro lentiviral expression vector.

Genes encoding PRRSV nsp1α, nsp1β, nsp2, nsp4, nsp5, nsp7, nsp8, nsp9, nsp10, nsp11, nsp12 and structural protein GP2, GP3, GP4, GP5, M and N proteins were amplified from infectious clone plasmid pBAC-SD16. All nsps were cloned into the pCAGGS-HA plasmid. The structural proteins were constructed into the pCAGEN plasmid. The PRRSV nsp4 truncated mutants were constructed and cloned into the pCMV-HA as previously described [38]. PRRSV nsp4 site mutation variants were also inserted into the pCAGGS-HA vector. All constructs were sequenced to verify their integrity. PCR primers are listed in Table 1.

### Immunoprecipitation and Western blotting

HEK293T cells were co-transfected pTRIP-GBP1-flag or the plasmids coding for its derivatives (1 μg/well) together with pCAGGS-nsp4-HA or its corresponding truncated mutants (1 μg/well). At 48 hpt, cells were collected and lysed using NP40 lysis buffer (containing 0.1 mM phenylmethylsulfonyl fluoride) for 30 min on ice. Cell lysates were centrifuged at 12 000 × *g* at 4 °C for 10 min to obtain clear supernatants. For the co-immunoprecipitation (Co-IP) assay, 100 μL Protein G Magbeads (GenScript, Nanjing, China) were incubated with 2 μg anti-flag or anti-HA antibody for 6 h at room temperature (RT), followed by incubation with cell supernatants for another 4 h at RT. After washing with phosphate buffered saline (PBS) three times, the beads were boiled with 2 X SDS loading buffer for 5 min at 100 °C.

The proteins were subjected to SDS-PAGE, and then transferred to a PVDF membrane. After blocking with 2.5% dry milk for 1 h at RT, the membranes were incubated with mouse anti-HA mAb at a dilution of 1:1000, rabbit anti-flag polyclonal antibody (pAb) at a dilution of 1:1000, mouse anti-α-tubulin mAb at a dilution of 1:5000 or mouse anti-Nsp4 pAb at a dilution of 1:1000 (produced in our lab) for 2 h at RT, respectively. After washing with PBST three times, the membranes were incubated with HRP-conjugated goat anti-mouse IgG or goat anti-rabbit IgG (1:2000) at RT for 1 h. After washing three times with PBST, the membranes were visualized by AMERSHAM ImageQuant 800 imaging system (Amersham Biosciences, Buckinghamshire, United Kingdom).

### Determination of PRRSV nsp4 cleavage effect

To investigate whether PRRSV infection affected GBP1 protein expression, Marc-145-GBP1-flag cells were infected with PRRSV at a MOI of 0.1. At 24, 36 and 48 hpi, cell samples were harvested for Western blotting analysis using anti-flag pAb. In another parallel experiment, Marc-145-GBP1-flag cells were infected with 0.1, 0.5 and 1.0 MOI of PRRSV for 36 h. Cells were then harvested for Western blotting analysis. Marc-145-GBP1-flag cells mock infected with PRRSV were used as the control group simultaneously.

To further determine whether nsp4 exerted a cleavage effect on GBP1, pCAGGS-nsp4-HA (1.0 μg/well) were co-transfected into HEK293T cells with pTRIP-Vector or pTRIP-GBP1-flag (0.5, 1.0, 1.5 μg/well). At 36 hpt, cells were harvested and subjected to Western blotting analysis by using anti-flag pAb.

### Statistical analysis

Statistical analysis was performed using GraphPad Prism 5.0 software (GraphPad Software, Inc., San Diego, USA). Two groups were compared by using the unpaired Student’s *t*-test, and multiple groups were compared by one-way analysis of variance (ANOVA). The data are presented as the mean ± SD. All experiments were performed at least three times. *P* < 0.05 was considered to indicate a statistically significant difference.

## Results

### Overexpression of GBP1 inhibits PRRSV infection and replication in vitro

**Figure 1:**
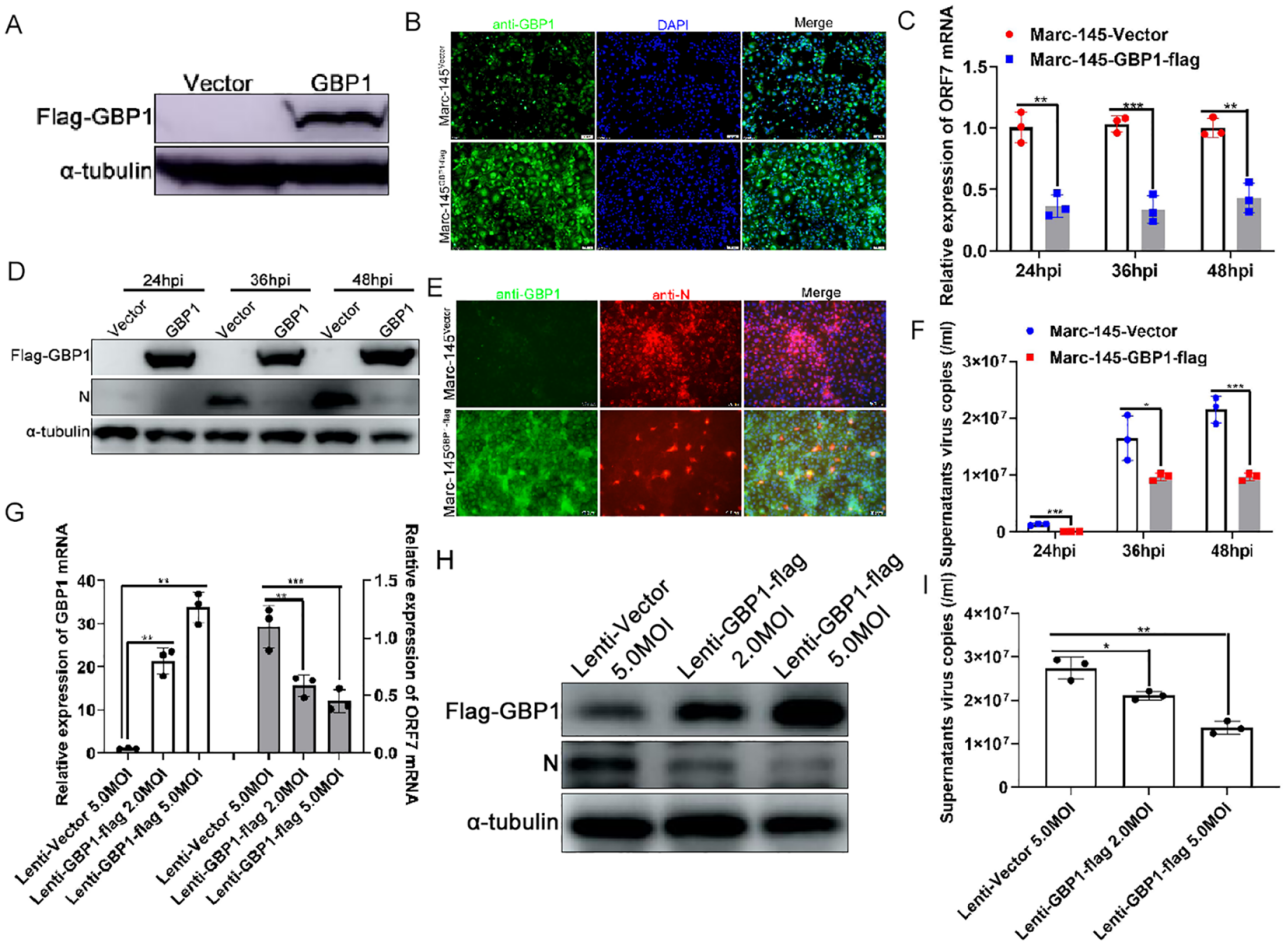
**Overexpression of GBP1 suppresses PRRSV replication in vitro.** **A** Western blotting verification of Marc-145 cells stably expressing Flag-GBP1. **B** Immunofluorescence assay identification of Marc-145-Vector and Marc-145-GBP1-flag recombinant cell lines using rabbit anti-GBP1 mAb and Alexa Fluor 488-conjugated goat anti-rabbit IgG (H&L). Marc-145-Vector and Marc-145-GBP1-flag cells were infected with a 0.1 MOI of PRRSV for 24, 36 and 48 h. Cells were harvested for the detection of (**C** and **F**) PRRSV ORF7 mRNA expression and viral copy numbers in the supernatant via qPCR, while **D** protein expression was detected via Western blotting. **E** Marc-145-Vector and Marc-145-GBP1-flag cells were infected with PRRSV (MOI = 0.1). Infected cells were assessed by incubation with anti-GBP1 and anti-PRRSV N antibodies, followed by incubation with Alexa Fluor 488-conjugated goat anti-rabbit IgG (H&L) and Alexa Fluor 594-conjugated goat anti-mouse IgG (H&L) antibodies, then samples were used for confocal microscopy observation. Porcine alveolar macrophages were inoculated with 2.0 and 5.0 MOI of recombinant lentivirus expressing GBP1 or 5.0 MOI of lenti-Vector for 24 h, followed by infection with 0.1 MOI of PRRSV. At 36 h post-infection, cells were harvested for **G** GBP1 and PRRSV ORF7 mRNA expression analysis via qPCR, and **H** for GBP1 and N protein expression analysis via Western blotting. **I** Supernatants were harvested for progeny viral copy detection via qPCR. GBP1, guanylate-binding protein 1; PRRSV, porcine reproductive and respiratory syndrome virus; ORF, open reading frame; qPCR, quantitative PCR.

To investigate the effect of GBP1 on PRRSV infection in vitro, Marc-145 cells stably expressing porcine GBP1 were first established by using a lentiviral expression system. Marc-145 cells were inoculated with pseudotyped virus with or without porcine GBP1, and selected with 8 μg/mL puromycin. The recombinant cell lines were identified via Western blotting and indirect IFA, respectively. Western blotting results showed that GBP1 was successfully expressed compared with the empty control group (Figure 1A). The IFA results showed that the fluorescence intensity of the GBP1-overexpressing group was notably increased compared with the control group (Figure 1B). These results revealed that Marc-145 cells stably expressing porcine GBP1 were successfully established, and the recombinant cell lines were named Marc-145-Vector and Marc-145-GBP1-flag, respectively.

Next, we explored the effect of GBP1 overexpression on PRRSV infection in Marc-145 cells. Marc-145-Vector or Marc-145-GBP1-flag cells were infected with 0.1 MOI of PRRSV. Cell samples were harvested at 24, 36 and 48 hpi, respectively, to determine the expression of PRRSV ORF7 mRNA and N protein. As shown in Figure 1C, compared with the control group, overexpression of GBP1 markedly decreased PRRSV ORF7 mRNA level at all three time points tested. Western blotting and IFA analysis of N protein expression showed that overexpression of GBP1 notably abrogated N protein expression (Figures 1D and E). Absolute qPCR detection of supernatant progeny virus copies showed that at all time points tested, supernatant viral copies were lower in the GBP1 overexpressing group than the control group (Figure 1F). As the natural target cells of PRRSV, the effect of GBP1 on PRRSV replication in PAMs was investigated as well. Since plasmid transformation efficiency is very low in PAMs, GBP1 was overexpressed in PAMs using the lentiviral expression system as described previously [18]. qPCR results suggested that GBP1 mRNA expression was notably up-regulated in a lentivirus dose-dependent manner compared with the control group (Figure 1G). Meanwhile, overexpression of GBP1 suppressed PRRSV replication in PAMs, as shown by the qPCR (Figure 1G) and Western blotting results (Figure 1H). Additionally, overexpression of GBP1 decreased supernatant viral copies in a dose-dependent manner as well (Figure 1I). Taken together, these results suggested that GBP1 is a PRRSV replication restrictive factor in both Marc-145 cells and PAMs.

### GBP1 disrupts the formation of actin filaments to suppress PRRSV replication and intercellular spread

**Figure 2:**
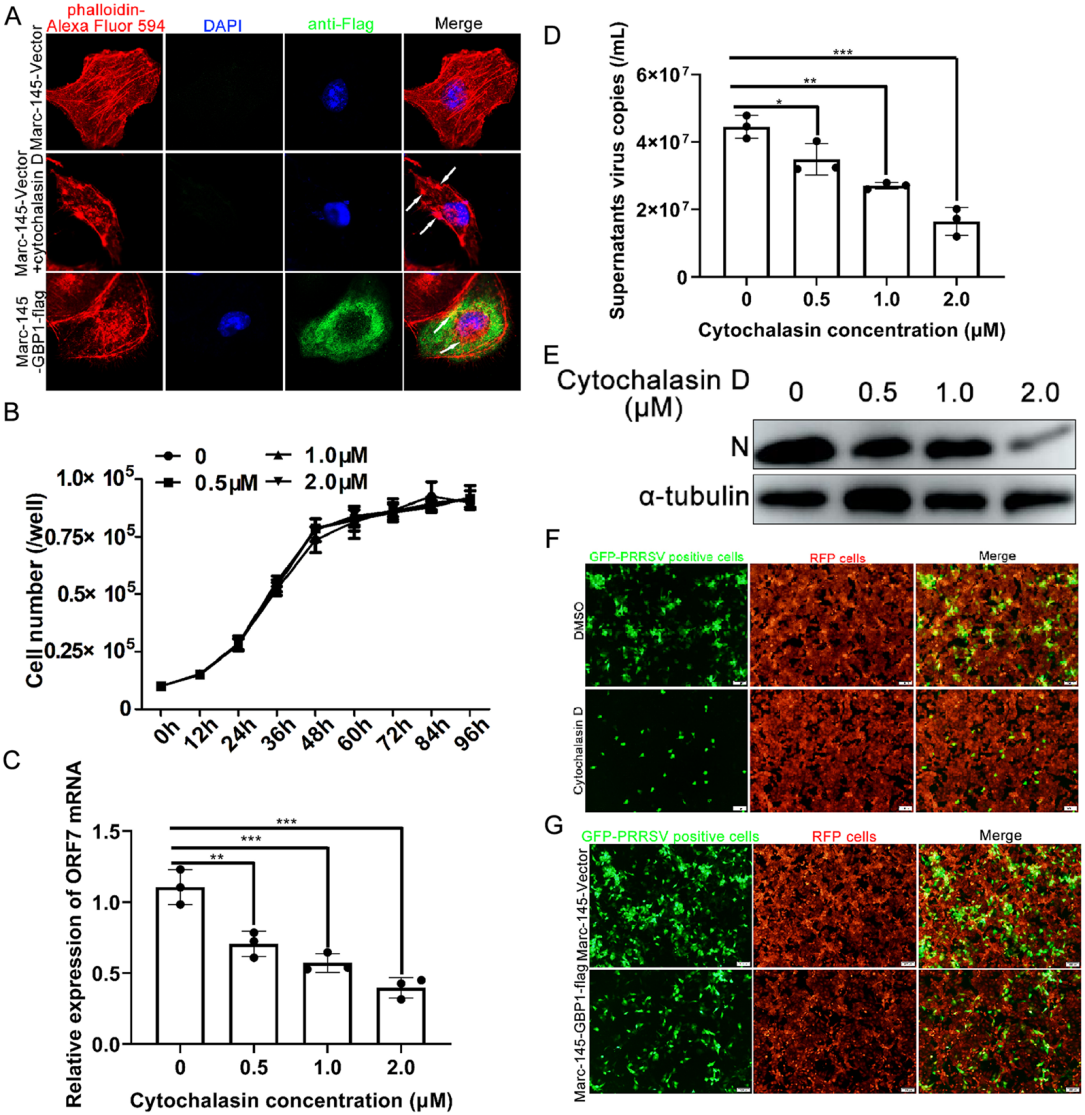
**Overexpression of GBP1 inhibits PRRSV replication by disrupting the stress fibers of the actin cytoskeleton.** **A** Marc-145-Vector or Marc-145-GBP1-flag cells were visualized using rabbit anti-flag pAb and Alexa Fluor 488-conjugated goat anti-rabbit IgG (H&L) antibodies, and actin was visualized using Alexa Fluor 546-labeled phalloidin antibody. As the control, Marc-145-Vector cells were treated with 2 μM cytochalasin D for 12 h, and actin was visualized using labeled phalloidin. **B** The growth curves of cells treatment with 0, 0.5, 1.0, 2.0 μM of cytochalasin were shown. Cells for each treatment were seeded at a concentration of 1 × 10^4^ cells/well and split daily for 4 consecutive days and cells were counted to determine cell number. **C**–**E** Marc-145 cells infected with 0.1 MOI of PRRSV were treated with 0, 0.5, 1.0, 2.0 μM cytochalasin D from 1 hpi onwards. At 36 hpi, cells were collected to determine the expression of ORF7 mRNA, progeny viral copies in the supernatant and N protein via quantitative PCR and Western blotting, respectively. **F** Marc-145 cells were infected with GFP-PRRSV (0.1 MOI). When ~60% of the cells were GFP positive, cells were sorted and used as donor cells to co-culture with Marc-145-RFP cells (red, target cells) for 2 h. Target cells were sorted and cultured with or without 2.0 μM cytochalasin D, and transmission was monitored by the fraction of GFP^+^RFP^+^ target cells at 36 hpi. **G** GFP-PRRSV-positive Marc-145 cells were sorted and used as donor cells for co-culture with Marc-145-GBP1-RFP cells for 2 h. Target cells were sorted and cultured, and transmission was monitored by the fraction of GFP^+^RFP^+^ target cells at 36 hpi. GBP1 guanylate-binding protein 1; PRRSV porcine reproductive and respiratory syndrome virus; hpi h post-infection; ORF open reading frame; GFP green fluorescent protein; RFP red fluorescent protein.

Studies have shown that hGBP1 can disturb the natural cytoskeletal structure by disrupting the formation of actin filaments through a direct interaction with the actin protein [39]. A natural and intact cytoskeletal structure is vital for efficient PRRSV replication [40]. This led to the speculation that GBP1-induced actin structural destruction may participate in PRRSV replication inhibition. To determine whether GBP1 could disrupt the microfilament structure to block efficient PRRSV infection, the formation of actin filaments in Marc-145-GBP1-flag cells and the control cells was investigated by confocal microscopy. In line with previously reported findings, GBP1 was located predominantly in the cytoplasm, in a granular structure (Figure 2A, bottom panel), and there was a very distinct microfilament structure in Marc-145-Vector cells (Figure 2A, top panel), while the actin filaments were impaired in Marc-145-GBP1-flag cells (Figure 2A, bottom panel). As the positive control, the actin specific inhibitor cytochalasin D (1.0 μM) markedly disrupted the normal fibrous structure of intracellular actin in Marc-145-Vector cells (Figure 2A, middle panel). To explore the effect of cytoskeletal proteins on PRRSV replication, Marc-145 cells were pre-treated with or without 0.5, 1.0 and 2.0 μM cytochalasin D for 12 h before transfection with a 0.1 MOI of PRRSV. The results showed that treatment with cytochalasin D abolished PRRSV infection of Marc-145 cells in a concentration-dependent manner (Figures 2C–E). However, within the concentrations used, cytochalasin D showed no significant effect on cell growth activity (Figure 2B). Additionally, a viral intercellular transmission assay based on the cell co-culture system showed that treatment with 2.0 μM cytochalasin D efficiently blocked PRRSV intercellular transmission in Marc-145 cells (Figure 2F). Furthermore, overexpression of GBP1 prohibited PRRSV intercellular spread as well (Figure 2G), which was similar to the effect of cytochalasin D treatment. However, PRRSV replication and intercellular spread was not completely abolished by overexpression of GBP1, indicating that the morphological change of actin is one of the molecular mechanisms by which GBP1 exerts anti-PRRSV activity.

### Knockdown of the expression of endogenous GBP1 promotes PRRSV replication

**Figure 3:**
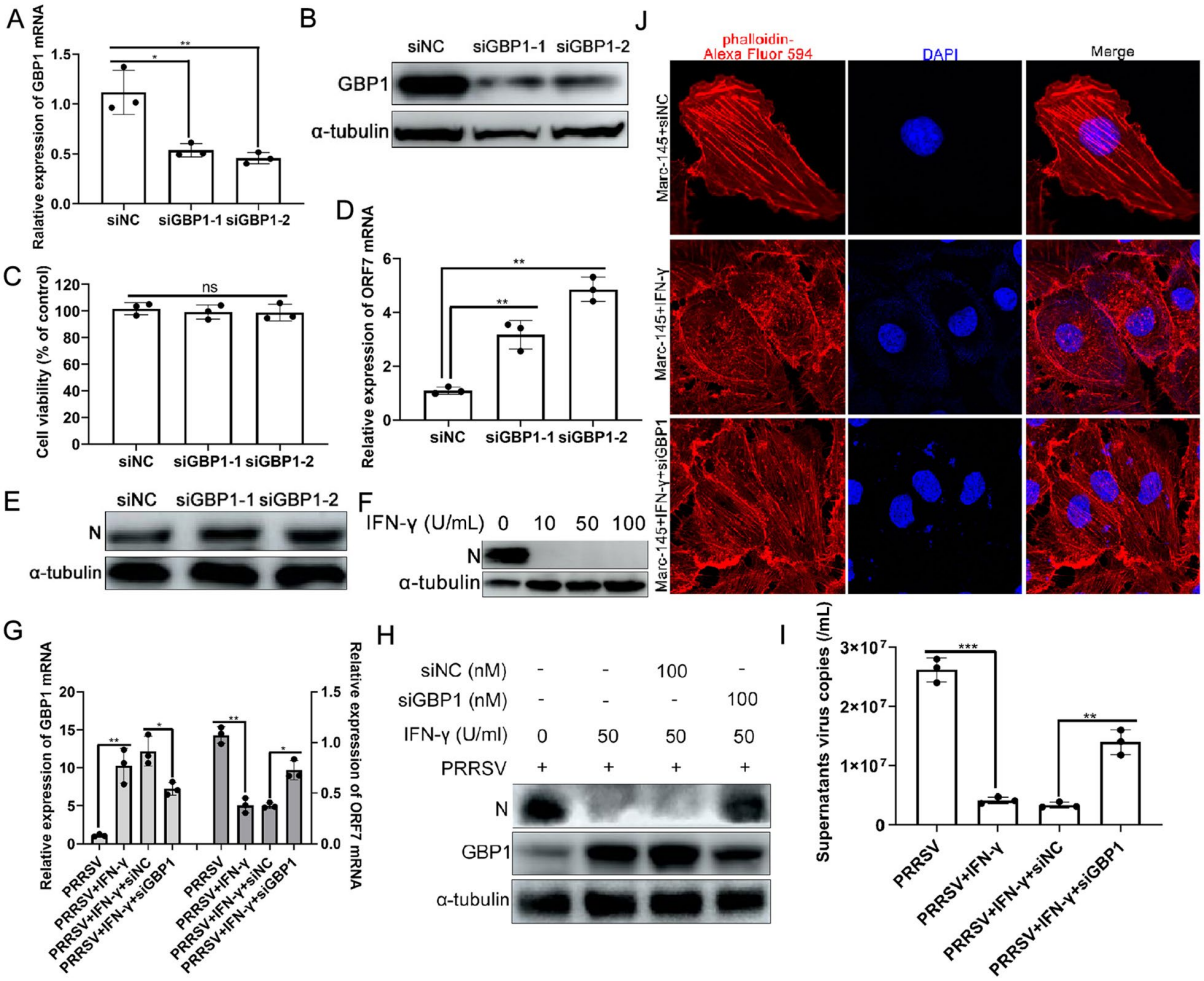
**Knockdown of endogenous GBP1 promotes PRRSV replication.** **A** and **B** Efficiency of endogenous GBP1 knockdown by siRNAs. PAMs transfected with 100 nM siGBP1 targeting different sites of GBP1 or siNC were harvested at 36 h post-transfection. The efficiency of GBP1 knockdown was assessed using **A** qPCR and **B** Western blotting. **C** Viability of PAMs transfected with siGBP1 or siNC was determined using a Cell Counting Kit-8 assay. **D** and **E** Knockdown of endogenous GBP1 expression enhances PRRSV replication. PAMs transfected with 100 nM siGBP1 were infected with a 0.1 MOI of PRRSV for 36 h. The expression of PRRSV ORF7 mRNA was assessed via **D** qPCR, and **E** N protein was assessed via Western blotting. **F** Marc-145 cells that were pretreated with or without IFN-γ (10, 50, 100 U/mL) for 12 h were inoculated with PRRSV at a MOI of 0.1. Cells were harvested at 36 h post-infection for the detection of N protein by using Western blotting. **G**–**I** GBP1 mediated the anti-PRRSV activity of IFN-γ. Marc-145 cells transfected with 100 nM siGBP1 or siNC for 12 h were treated with or without 50 U/mL IFN-γ for another 12 h, followed by infection with a 0.1 MOI of PRRSV for 24 h. The expression of PRRSV ORF7 mRNA, supernatant viral copies and N protein were analyzed via **G** and **I** qPCR and **H** Western blotting, respectively. **J** GBP1 was necessary for the regulation of the remodeling of the actin cytoskeleton induced by IFN-γ. Marc-145 cells were transfected with either siNC or siGBP1 as indicated. After 12 h, the cells were either left untreated or treated with IFN-γ (100 U/mL) for 24 h and subsequently stained for actin. GBP1 guanylate-binding protein 1; PRRSV porcine reproductive and respiratory syndrome virus; siRNA small interfering RNA; PAM porcine alveolar macrophage; NC negative control; qPCR quantitative PCR; ORF open reading frame; IFN interferon.

Since overexpression of GBP1 blocked PRRSV replication in vitro, it was next investigated whether endogenous GBP1 played a role during PRRSV infection. PAMs were transfected with either siNC or siGBP1 at a final concentration of 100 nM, and the expression of endogenous GBP1 was assessed to validate the silencing efficiency of the siRNAs. The expression levels of GBP1 mRNA and protein were both significantly reduced by siGBP1, but not siNC (Figures 3A and B). There was no significant difference in cell viability between GBP1 and scrambled siRNA-treated cells based on the CCK-8 cell viability assay (Figure 3C). PAMs were transfected with 100 nM siNC, siGBP1-1 or siGBP1-2 for 12 h and then infected with a 0.1 MOI of PRRSV for another 36 h, following which the efficiency of PRRSV infection was assessed. As shown in Figures 3D and E, knockdown of endogenous GBP1 promoted PRRSV ORF7 mRNA and N protein expression. These results suggested that endogenous GBP1 plays an important inhibitory role during PRRSV replication.

Next, the role of GBP1 in mediating the effect of IFN-γ on PRRSV infection was investigated. First, the effects of IFN-γ on PRRSV infection in Marc-145 cells were determined. As shown in Figure 3F, IFN-γ obviously suppressed PRRSV replication in a concentration-dependent manner in Marc-145 cells. Next, the effect of IFN-γ on GBP1 expression in Marc-145 cells was detected. Upregulation of GBP1 was observed after treatment with IFN-γ in Marc-145 cells (Figure 3G). To further determine whether GBP1 mediated the antiviral activity of IFN-γ, Marc-145 cells transfected with siGBP1 or siNC for 12 h were treated with 50 U/mL IFN-γ for another 12 h, and then inoculated with a 0.1 MOI of PRRSV. Western blotting and absolute qPCR results showed that silencing of endogenous GBP1 decreased the anti-PRRSV activity of IFN-γ (Figures 3G and I). Previous reports reflected that IFN-γ-induced GBP1 is a novel actin remodeling factor [39]. Thus, the effects of IFN-γ on actin morphology in Marc-145 cells were detected. Consistent with previous studies, the actin cytoskeleton exhibited a distorted structure in the presence of siNC combined with 100 U/mL IFN-γ, but this effect was largely abrogated with the siGBP1 (Figure 3J). These results suggested that the antiviral activity of IFN-γ mediated by GBP1 may be exerted by the morphological alteration of normal actin induced by GBP1.

### GTPase activity and dimerization of GBP1 are required for the antiviral activity of GBP1 during PRRSV infection

**Figure 4:**
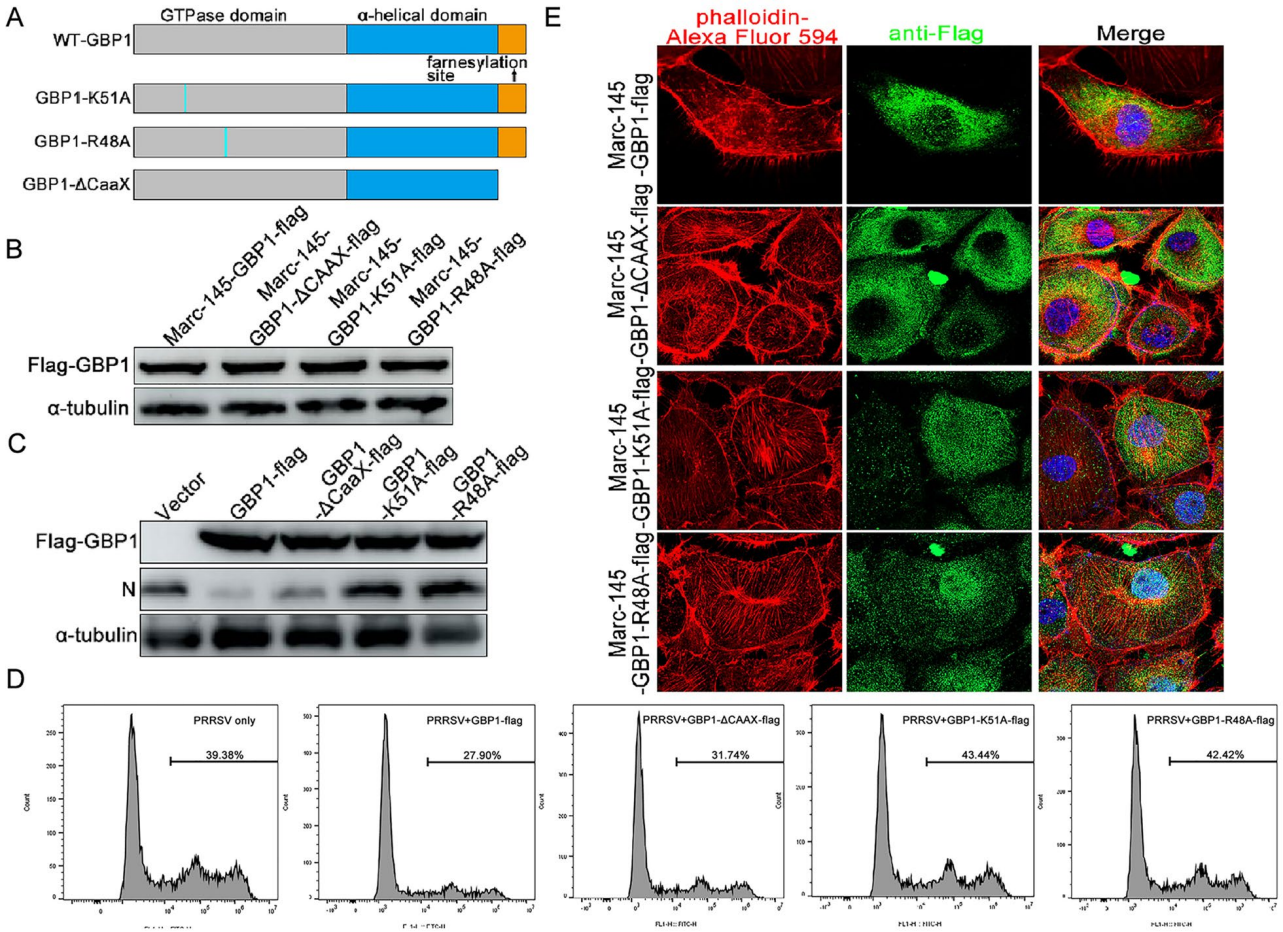
**GTPase activity and dimerization of GBP1 is necessary for the inhibition of PRRSV replication.** **A** Map of the full-length and three site-specific mutants of GBP1. **B** Western blotting verification of Marc-145 cells stably expressing GBP1-ΔCAAX, -K51A, -R48A using rabbit anti-flag pAb. Marc-145-Vector, -GBP1-flag, -GBP1-ΔCAAX-flag, -GBP1-K51A-flag or -GBP1-R48A-flag were infected with GFP-PRRSV at a MOI of 0.1. At 36 h post-infection, cells were harvested for the **C** detection of N protein expression via Western blotting (**C**), and **D** for GFP-PRRSV-positive cell analysis via flow cytometry. **E** Marc-145-GBP1-flag, -GBP-ΔCAAX-flag, -GBP1-K51A-flag or -GBP1-R48A-flag were used for the detection of GBP1 using anti-flag pAb. Actin was visualized with fluorescently labeled phalloidin. GBP1, guanylate-binding protein 1; PRRSV, porcine reproductive and respiratory syndrome virus; GFP, green fluorescent protein.

Structural hallmarks of GBP1 are a large globular α/β-domain harboring the GTPase activity, an elongated C-terminal region arranged in an α-helical structure with unique features and a CAAX motif at the C-terminal end that causes isoprenylation of GBP1 [41, 42]. The R48 and K51 residues are important for its GTPase activity of human GBP1 [43]. Mutation of the cysteine residue in the CAAX box prevents GBP1 isoprenylation and anchoring into membranes [44]. Thus, three GBP1 mutants, including GBP1-ΔCAAX, -K51A and -R48A, were constructed (Figure 4A), and Marc-145 cells stably expressing these proteins were selected by using puromycin. As shown in Figure 4B, Western blotting results confirmed that all three mutant cell lines were successfully established, and named Marc-145-GBP1-ΔCAAX-flag, Marc-145-GBP1-K51A-flag and Marc-145-GBP1-R48A-flag. To determine whether GTPase activity, dimerization and membrane localization were responsible for the inhibition of PRRSV infection, the GBP1-wt and mutant Marc-145 cell lines were infected with 0.1 MOI of PRRSV or GFP-PRRSV. Western blotting results showed that overexpression of GBP1 notably reduced PRRSV infection, while mutation at the K51 or R48 site, particularly at the R48 site, completely reversed the antiviral effect of GBP1. However, the deletion of the CAAX motif revealed similar antiviral effects as GBP1-wt, although slightly weaker (Figure 4C). Flow cytometry analysis of GFP-PRRSV-positive Marc-145 cell percentage exhibited similar phenomenon (Figure 4D).

Since GBP1-wt disrupted the actin stress fibers (Figure 2A), next we assessed whether GTPase activity was necessary for actin remodeling. Confocal microscopy analysis revealed that actin stress fibers were selectively impaired in Marc-145 cells expressing GBP1-wt, but were not notably affected in cells expressing GBP1-K51A and -R48A (Figure 4E). Previous studies have shown that GBP1-CAAX mutation is not farnesylated and does not associate with the plasma membrane [45]. A mutant with a deleted CAAX motif was used to investigate the potential further influence of GBP-1 in the remodeling of the cytoskeleton. The staining of the actin cytoskeleton in GBP1-CAAX expressing cells showed that the lack of GBP-1 membrane association did not abrogate its effect on the remodeling of the actin cytoskeleton (Figure 4E). Considering that the normal physiological morphology of actin is necessary for effective PRRSV replication, we speculated that GBP1-K51A and -R48A failed to suppress PRRSV replication, which could at least be partially attributed to the lack of their ability to induce morphological changes to actin. However, GBP1-CAAX mutant still disturbed PRRSV replication, indicating that the plasma membrane association is not required in this process.

### GBP1 is a host cellular factor that interacts with PRRSV nsp4 protein

**Figure 5:**
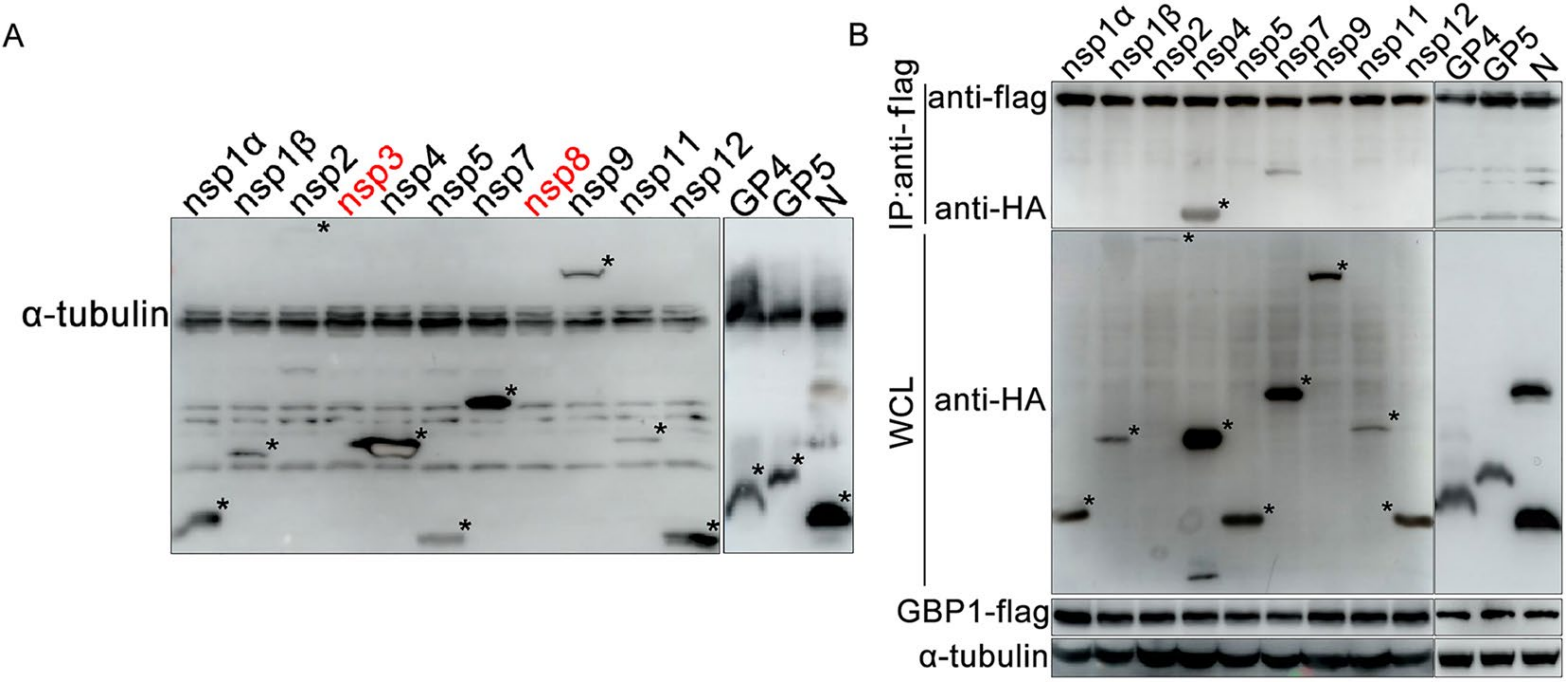
**Screening of PRRSV proteins that interact with GBP1.** **A** Western blotting identification of the expression of PRRSV-relevant proteins. HEK293T cells were transfected with the plasmids expressing HA-tagged proteins of PRRSV. The cells were harvested at 36 h post-transfection and subjected to Western blotting analysis. **B** Screening of PRRSV proteins that interact with GBP1. HEK293T cells were co-transfected with the plasmids expressing HA-tagged proteins of PRRSV and Flag-GBP1 for 48 h. Cell samples were lysed and co-immunoprecipitation was performed using anti-flag pAb. GBP1, guanylate-binding protein 1; PRRSV, porcine reproductive and respiratory syndrome virus.

Previous studies have shown that GBP1 may inhibit virus replication via interacting with viral non-structural proteins [28–30]. To further explore the mechanism underlying the anti-PRRSV activity of GBP1, whether GBP1 suppressed PRRSV replication via interacting with PRRSV proteins was investigated. Firstly, eukaryotic expression plasmids expressing PRRSV nsp1α, 1β, 2, 3, 4, 5, 7, 8, 9, 11, 12, GP2, GP3, GP4, GP5, M and N were constructed and transfected into HEK293T cells. As shown in Figure 5A, PRRSV nsp1α, 1β, 2, 4, 5, 7, 9, 11, 12, GP4, GP5 and N proteins were successfully expressed, while nsp3, nsp8, GP2, GP3 and M proteins were not expressed (data not shown). Of note, nsp2 expression was very low (Figure 5A).

Then, HEK293T cells were transfected with pTRIP-GBP1-flag together with pCAGGS-HA-nsp1α, 1β, 2, 4, 5, 7, 9, 11, 12, GP4, GP5 and N. Transfection products were subjected to co-IP assay with anti-flag mAb at 48 hpt to screen the potential proteins that might interact with each other. Western blotting results indicated that PRRSV NSPs and GPs were successfully expressed in HEK293T cells (Figure 5B, input panels). Co-IP results showed that GBP1 interacted specifically with PRRSV nsp4 and nsp7, but not other NSPs or GPs (Figure 5B).

**Figure 6:**
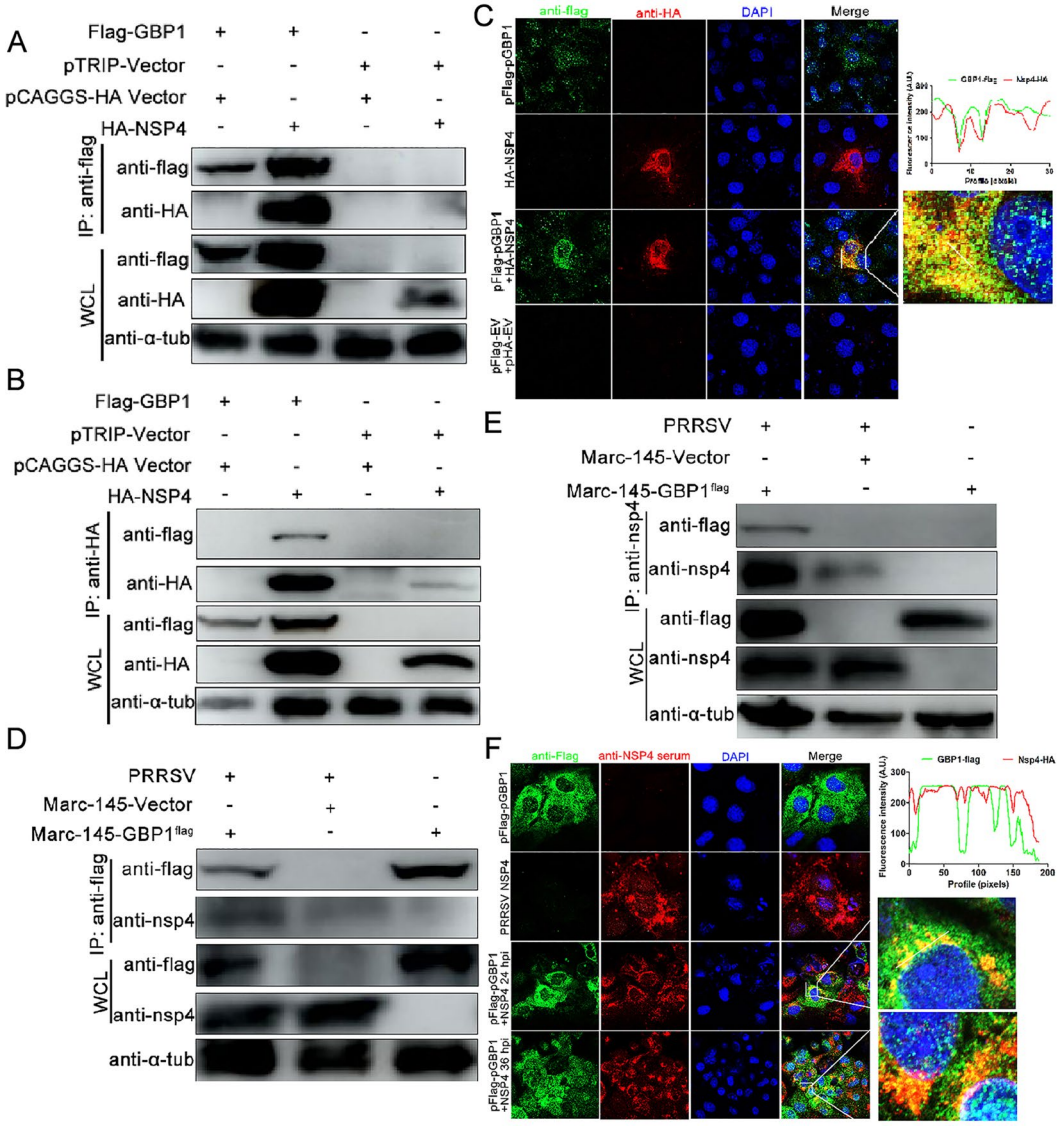
**PRRSV nsp4 interacts with GBP1.** **A** and **B** Exogenous co-IP assay in HEK293T cells. HEK293T cells were co-transfected with Flag-GBP1 (1 μg) with or without HA-nsp4 (1 μg). At 48 h post-transfection, cells were harvested for co-IP analysis using Protein G Magbeads coated with **A** rabbit anti-flag pAb (green) or **B** mouse anti-HA mAb (red). **C** Expression plasmids Flag-GBP1 and HA-nsp4 were co-transfected into HEK293T cells and then subjected to a confocal assay. **D** and **E** Marc-145-Vector or Marc-145-GBP1-flag cells were infected with PRRSV at a MOI of 1.0 for 48 h. Cells were harvested and subjected to co-IP assay using beads coated with **D** rabbit anti-flag pAb or **E** mouse anti-nsp4 pAb. **F** Marc-145-Vector or Marc-145-GBP1-flag cells infected with PRRSV (MOI = 0.1) for 36 h were fixed and stained with rabbit anti-flag pAb (green) and mouse anti-nsp4 pAb (red), and the nuclei were stained with DAPI. Fluorescent images were acquired with a confocal laser scanning microscope. Scale bar, 10 μm. GBP1 guanylate-binding protein 1; PRRSV porcine reproductive and respiratory syndrome virus; nsp4 non-structural protein 4; co-IP co-immunoprecipitation.

To further confirm the interaction between nsp4 and GBP1, a co-IP assay was further conducted using anti-flag mAb together with protein G Magbeads. Co-IP results suggested that Flag-GBP1 interacted with HA-nsp4 after incubation with an anti-flag mAb and protein G Magbeads (Figure 6A). Furthermore, HA-nsp4 was shown to be co-immunoprecipitated with Flag-GBP1 after incubation with an anti-HA mAb and protein G Magbeads (Figure 6B). The confocal microscopy illustrated the colocalization between GBP1 and nsp4 in the cytoplasm of flag-GBP1 and HA-nsp4 co-transfected HEK293T cells (Figure 6C). For the interaction between GBP1 and nsp7, nsp7 was co-immunoprecipitated with GBP1 when protein G Magbeads were coated with anti-flag mAb, however, nsp7 was not co-immunoprecipitated with GBP1 when the Magbeads were coated with anti-HA mAb (data not shown). Thus, nsp4 was chosen for the following experiments.

Next, the interaction between GBP1 and PRRSV nsp4 protein in PRRSV-infected Marc-145 cells was examined. Marc-145-GBP1-flag cells were mock infected or infected with PRRSV at a MOI of 0.1. Cells were collected for co-IP detection with lysates of PRRSV mock infected or infected cells and Protein G Magbeads conjugated with antibodies against flag tag. GBP1 was immunoprecipitated with PRRSV nsp4 protein in PRRSV-infected cell lysates (Figure 6D). When co-IP was conducted using mouse anti-nsp4 pAb, GBP1 was also pulled down by nsp4 (Figure 6E). Then, Marc-145-GBP1-flag cells were mock infected or infected with PRRSV and co-IP was conducted at 24 and 36 hpi, respectively. Confocal images revealed that GBP1 co-localized with nsp4 in the cytoplasm of the PRRSV-infected Marc-145-GBP1-flag cells (Figure 6F), as indicated by the yellow fluorescence in the merged image. Collectively, these data suggested that GBP1 specifically interacted with PRRSV nsp4.

### Both K51 and R48 sites are critical for the GBP1-nsp4 interaction

**Figure 7:**
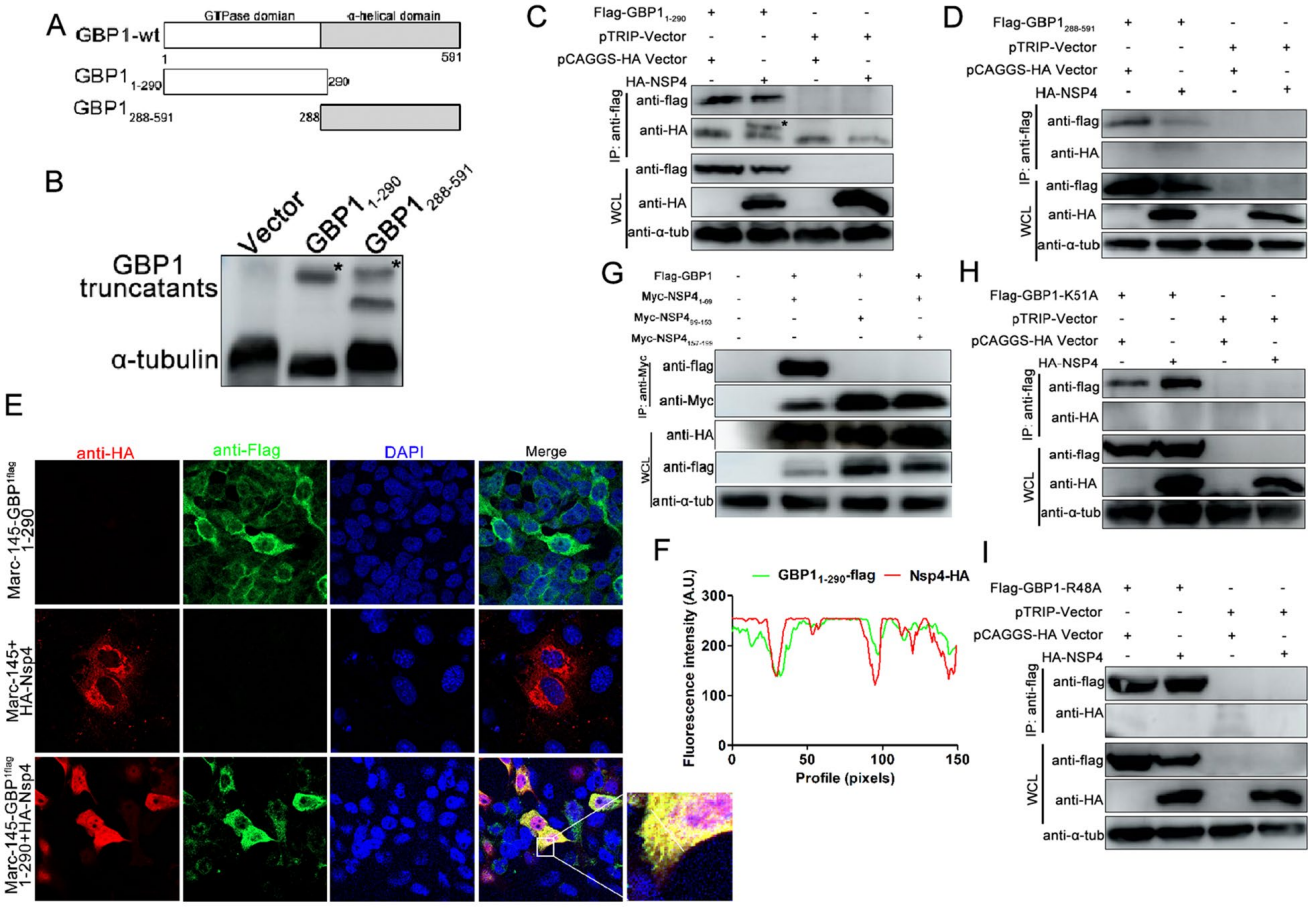
**GTPase activity of GBP1 is essential for GBP1-nsp4 interaction.** **A** Schematic representation of the protein domains of GBP1. **B** Expression of truncated mutants of GBP1. GBP1 truncated mutants Flag-GBP1_1-290_ and Flag-GBP1_288-591_ or empty vector were transfected into HEK293T cells. Cells were collected and analyzed using rabbit anti-flag pAb. **C** The N-terminal region of GBP1 interacted with nsp4. HEK293T cells were co-transfected with Flag-GBP1_1-290_ and full-length HA-nsp4. Cells were harvested and subjected to co-IP analysis using Protein G Magbeads coated with rabbit anti-flag pAb. **D** The C-terminal of GBP1 did not interact with PRRSV nsp4. Flag-GBP1_288-591_ and HA-nsp4 expression plasmids were co-transfected into HEK293T cells for 48 h. Cells were used for co-IP analysis using beads coated with rabbit anti-flag pAb. **E** The Marc-145-GBP1_1–290_-flag cell line was transfected with plasmid HA-nsp4, and a confocal assay was performed. Scale bar, 10 μm. **F** Colocalization analysis corresponding to Flag-GBP1_1–290_ and HA-nsp4. **G** The N-terminal domain of nsp4 interacted with GBP1. HEK293T cells were co-transfected with Myc-nsp4_1–69_ and Flag-GBP1. Cells were harvested for co-IP analysis using beads coated with mouse anti-Myc mAb. **H** and **I** HEK293T cells were co-transfected with Flag-GBP1-K51A or -R48A together with HA-nsp4, respectively. At 24 h post-transfection, cell lysates were collected for co-IP detection with beads conjugated with rabbit anti-flag pAb. GBP1, guanylate-binding protein 1; nsp4 non-structural protein 4; co-IP co-immunoprecipitation; PRRSV porcine reproductive and respiratory syndrome virus.

To define the key domain in GBP1 that determines its interaction with nsp4 protein, two GBP1 truncated mutants were generated, i.e., pTRIP-GBP1_1-290_-flag and pTRIP-GBP1_288-591_-flag (Figure 7A). Plasmids expressing truncated proteins of GBP1 were transfected into HEK293T cells, and cell lysates were used for Western blotting to confirm the expression of truncated proteins (Figure 7B). Then, these plasmids were co-transfected with HA-nsp4 into HEK293T cells, and subjected to co-IP assay. Co-IP results showed that the truncated mutants Flag-GBP1_1-290_ could bind to HA-nsp4 (Figure 7C). Nevertheless, the Flag-GBP1_288-591_ region did not interact with PRRSV nsp4 protein (Figure 7D). Additionally, confocal analysis showed that Flag-GBP1_1-290_ co-localized with HA-nsp4 primarily in the cytoplasm (Figures 7E and F). A reverse co-IP using mouse anti-Myc mAb was also performed. The results indicated that Flag-GBP1 co-immunoprecipitated with Myc-nsp4_1-69_ mutants (Figure 7G). These results suggested that the N-terminal domain of both GBP1 and nsp4 are critical for the nsp4-GBP1 interaction.

Since K51 and R48 of GBP1 are essential for GTPase activity and anti-PRRSV activity (Figure 4), whether K51 and R48 were required for nsp4-GBP1 interaction was further determined. Plasmids expressing Flag-GBP1-K51A or Flag-GBP1-R48A were co-transfected into HEK293T cells with HA-nsp4. At 48 hpt, cells were harvested and subjected to co-IP. However, neither Flag-GBP1-R48A nor Flag-GBP1-K51A pulled down HA-nsp4 (Figure 7H and I). These results suggested that GBP1 GTPase activity is crucial for the nsp4-GBP1 interaction.

### PRRSV infection cleaves GBP1, which is dependent on the 3CLSP catalytic activity of nsp4

**Figure 8:**
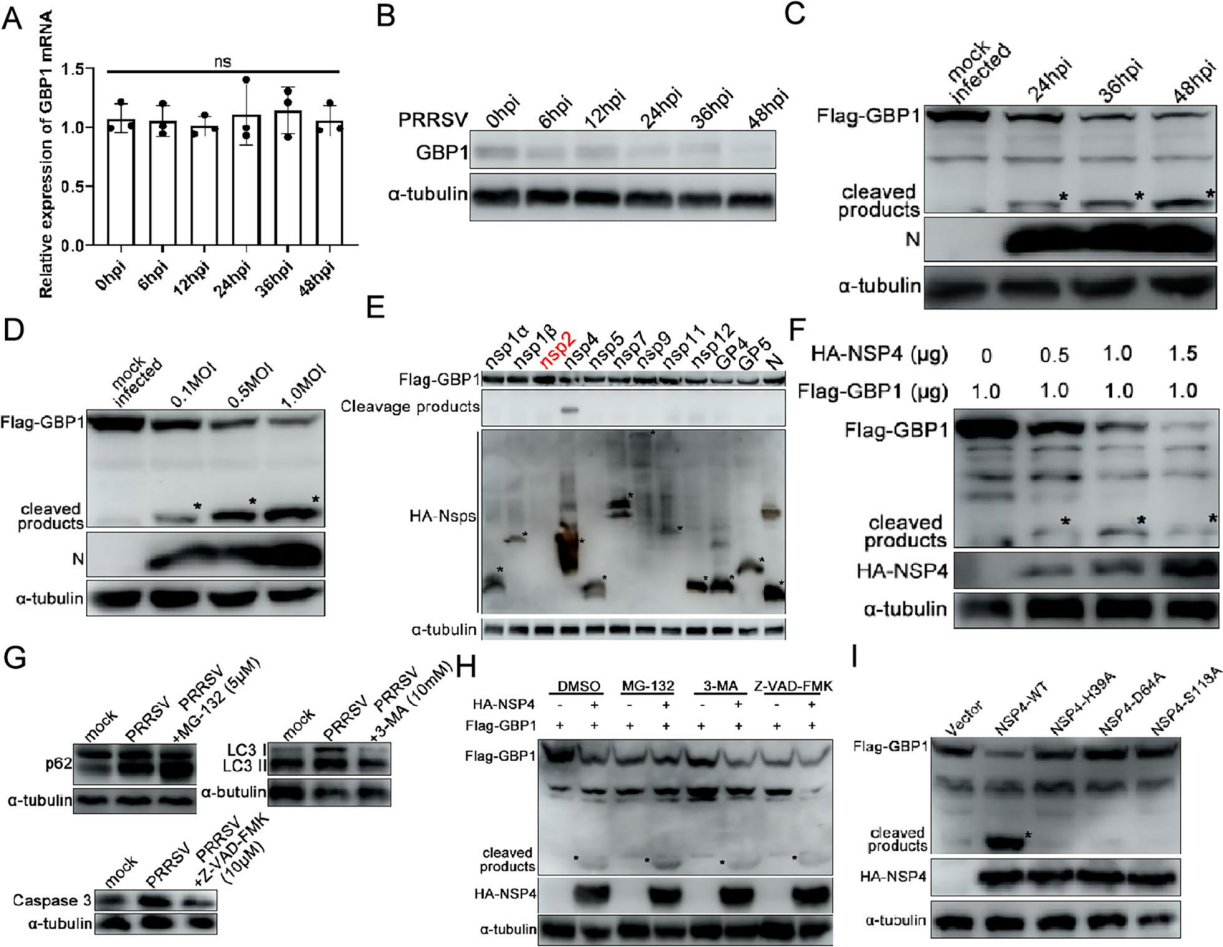
**PRRSV nsp4 cleaves GBP1 by its 3C-like serine protease activity.** **A** Porcine alveolar macrophages were infected with 0.1 MOI of PRRSV. Cells were collected at 0, 6, 12, 24, 36, 48 hpi to determine the expression of GBP1 mRNA via quantitative PCR and the expression of GBP1 protein via Western blot (**B**). **C** Marc-145-GBP1-flag cells were infected with 0.1 MOI of PRRSV. At 12, 24 and 36 hpi, cells were harvested for Western blot analysis to detect GBP1 and N protein expression. **D** Marc-145-GBP1-flag cells were infected with PRRSV at a MOI of 0.1, 0.5 and 1.0. At 48 hpi, cells were collected and subjected to Western blotting to detect GBP1 and N protein expression. **E** The effect of PRRSV proteins on GBP1 expression. HEK293T cells were co-transfected with the plasmids expressing Flag-GBP1 and HA-tagged PRRSV proteins. The cells were harvested at 48 hpt and subjected to Western blotting. **F** HEK293T cells were co-transfected with 1.0 μg Flag-GBP1 and different concentrations (0, 0.5, 1.0 or 1.5 μg) of HA-nsp4. Cells were collected at 48 hpt and assessed via Western blotting. **G**) Marc-145 cells mock infected or infected with PRRSV (0.1MOI) were treated with or without MG-132 (5 μM), 3-MA (10 mM) or Z-VAD-FMK (10 μM). At 36 hpi, cells were harvested for Western blot analysis. **H** The role of cellular protein degradation pathways in the reduction of GBP1 was assessed. HEK293T cells were co-transfected with Flag-GBP1 and HA-Nsp4. At 12 hpt, cells were treated with DMSO control, proteasome inhibitor MG-132 (5 μM), autophagy inhibitor 3-MA (10 mM) or the lysosome inhibitor Z-VAD-FMK (10 μM) for 24 h. The expression of GBP1 and nsp4 were examined by Western blotting. **I** Effect of the HA-nsp4 and its protease defective mutants on GBP1 expression. HEK293T cells were co-transfected with Flag-GBP1 together with HA-nsp4 or its protease defective mutants expression plasmids for 48 h, followed by Western blot analysis. GBP1 guanylate-binding protein 1; nsp4 non-structural protein 4; PRRSV porcine reproductive and respiratory syndrome virus; hpi hours post-infection; hpt hours post-transfection.

To determine whether PRRSV infection affects GBP1 expression, the mRNA levels of GBP1 in PAMs infected with PRRSV at different time points were detected. As shown in Figure 8A, PRRSV infection did not markedly affect the mRNA levels of GBP1. Next, the protein levels of GBP1 in PAMs upon PRRSV infection were analyzed. PRRSV infection also markedly dereased the protein level of GBP1 (Figure 8B). The next, Marc-145-GBP1-flag cells were mock infected or infected with 0.1 MOI of PRRSV, at 24, 36 and 48 hpi, the protein level of GBP1 was analyzed via Western blotting using anti-flag pAb to investigate the effect of PRRSV infection on GBP1 protein expression. As shown in Figure 8C, GBP1 protein expression gradually decreased with the progression of PRRSV infection. Furthermore, a small cleavage band of GBP1 was observed during PRRSV infection (Figure 8C). To further confirm the aforementioned results, Marc-145-GBP1-flag cells were infected with 0.1, 0.5 and 1.0 MOI of PRRSV. Cells were harvested at 36 hpi for the detection of GBP1 protein expression. In accordance with the aforementioned results, PRRSV infection reduced GBP1 protein expression in a dose-dependent manner, and obvious cleavage bands were observed simultaneously (Figure 8D). To screen the proteins of PRRSV responsible for the reduction of GBP1, HEK293T cells were co-transfected with HA-PRRSV proteins together with Flag-GBP1 for 36 h, and then the cell samples were harvested for Western blotting analysis. The results showed that GBP1 was notably cleaved following co-transfection of HA-nsp4 with Flag-GBP1, and no obvious cleavage band was observed in other co-transfection combinations (Figure 8E). These results indicated that PRRSV infection downregulates GBP1 expression by cleaving it, and nsp4 is likely to play a critical role.

To further verify the cleavage effect of nsp4 on GBP1, HEK293T cells were transfected with a constant dose of Flag-GBP1 together with increasing doses of HA-nsp4 for 36 h, and then cell samples were harvested for Western blotting analysis. A small cleavage band of GBP1, which was identical to the cleaved GBP1 in PRRSV-infected cells (Figure 8C), could only be observed in cells overexpressing nsp4 (Figure 8F). However, the Flag-GBP1 co-transfection with an empty vector did not affect the expression of GBP1 (Figure 8F), further confirming that PRRSV nsp4 is responsible for the cleavage of GBP1.

To assess the involvement of the lysosomal, proteasomal and caspase pathways in PRRSV-induced reduction of GBP1, Marc-145-GBP1-flag cells were pre-treated with DMSO, proteasome inhibitor MG-132 (5 μM), autophagy inhibitor 3-MA (10 mM), and the caspase inhibitor (Z-VAD-FMK, 10 μM) for 12 h, followed by infection with a 0.1 MOI of PRRSV, and the protein expression levels of GBP1 were detected by Western blotting. MG-132, 3-MA, and Z-VAD-FMK treatment enhanced PRRSV induced p62 activation, suppressed PRRSV induced LC3 and caspase 3 activation, respectively, demonstrating that these inhibitors worked well (Figure 8G). Nevertheless, PRRSV infection-mediated cleavage of GBP1 was not affected by treatment with the proteasome inhibitor MG-132, lysosome inhibitor 3-MA, or caspase inhibitor Z-VAD-FMK (Figure 8H), revealing that the cleavage of GBP1 by PRRSV infection was not affected by treatment with those inhibitors, and the cleavage of GBP1 by nsp4 was not associated with the cellular lysosome, proteasome or autophagy pathways.

PRRSV nsp4 is a member of a relatively rare group of proteolytic enzymes, the 3CLSPs, which contain the canonical catalytic triad of His39-Asp64-Ser118 and three domains located at amino acids 1–69, 89–153 and 157–199 [46, 47]. The interaction between GBP1 and PRRSV nsp4 suggested that nsp4 may participate in the cleavage of GBP1 via its 3CLSP catalytic activity. Thus, whether nsp4 protease activity was responsible for the cleavage of GBP1 was determined. Three nsp4 single-point mutant plasmids were constructed, including Nsp4-H39A, Nsp4-D64A and Nsp4-S118A. Any mutation to these amino acids will abolish nsp4 protease activity [47]. These mutant plasmids were co-transfected into HEK293T cells with Flag-GBP1. No obvious cleavage band was detected in cells co-transfected with Flag-GBP1 and HA-nsp4-H39A, HA-nsp4-D64A or HA-nsp4-S118A, except in the Flag-GBP1 and HA-nsp4 co-transfection group (Figure 8I). These results suggested that the protease activity of nsp4 is responsible for the cleavage of GBP1.

### The E338 site on GBP1 is necessary for the nsp4-mediated cleavage effect

**Figure 9:**
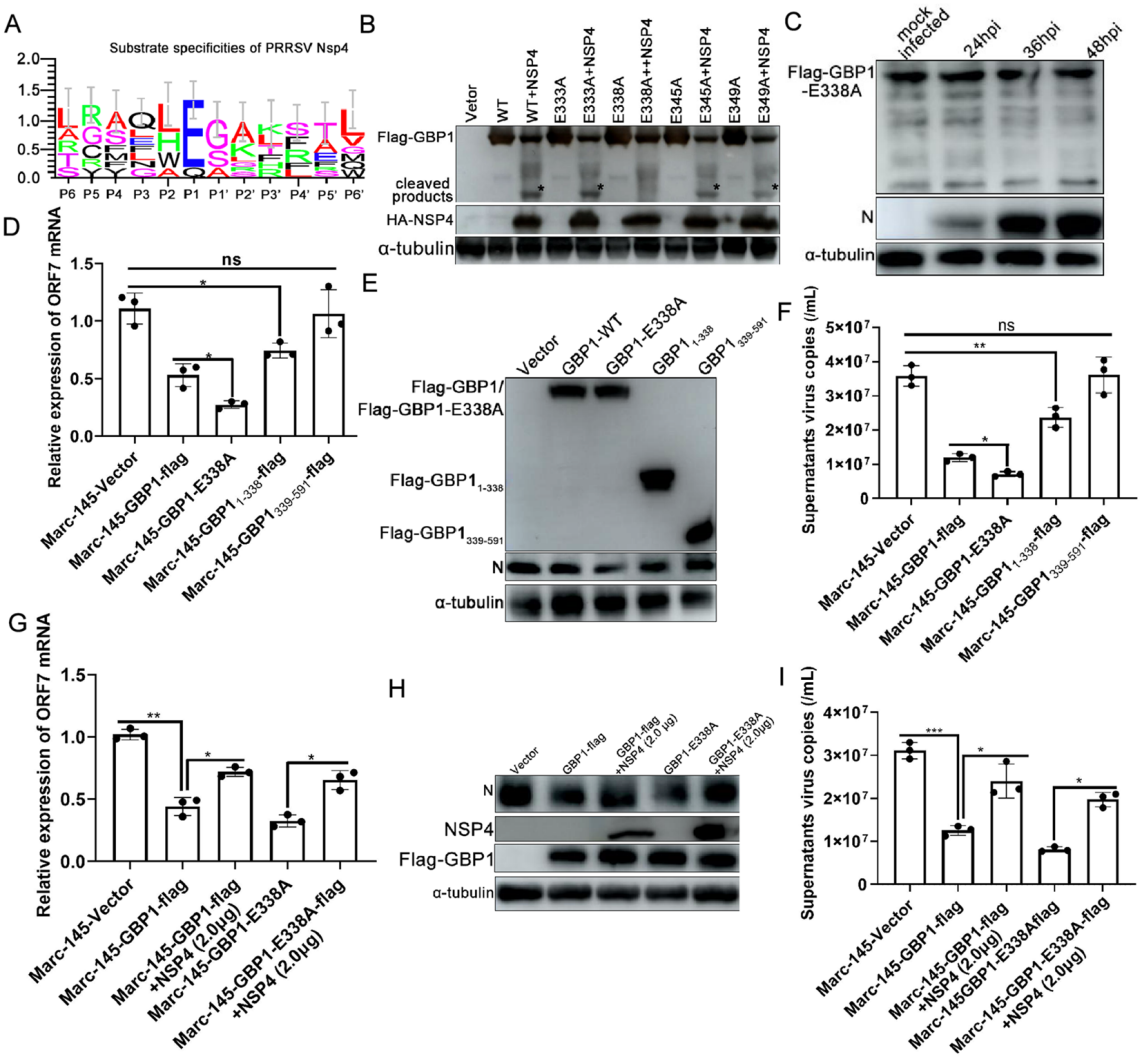
**PRRSV nsp4 cleaves GBP1 at E338 and this cleavage impairs the antiviral activity of GBP1.** **A** Sequence logo of the polyprotein junctions cleaved by PRRSV nsp4 from different strains. An amino acid sequence logo of the substrate was generated by using WebLogo. **B** HEK-239 T cells were co-transfected with Flag-GBP1, -GBP1 mutants and HA-nsp4. At 48 h post-transfection, cell lysates were prepared and analyzed by Western blotting. **C** Marc-145-GBP1-E338A-flag cells were mock infected or infected with 0.1 MOI of PRRSV. Cells were harvested at 24, 36, 48 hpi and subjected to Western blot analysis. **D**–**F** Mutation of nsp4 cleavage site enhanced the antiviral activity of GBP1. Marc-145-Vector, -GBP1-flag and -GBP1-E338A-flag cells were treated with 0.1 MOI of PRRSV. Cells were harvested at 36 h post-infection to determine PRRSV ORF7 mRNA and supernatant viral copies via qPCR, and the expression of N protein was determined by Western blotting. **G**–**I** Marc-145-GBP1-flag cells were transfected with 1.0 or 2.0 μg nsp4 expression plasmids for 12 h. Then, cells were inoculated with 0.1 MOI of PRRSV. Cells were harvested, PRRSV ORF7 mRNA and supernatant viral copies were detected by qPCR, N protein was detected by Western blotting. GBP1 guanylate-binding protein 1; nsp4 non-structural protein 4; PRRSV porcine reproductive and respiratory syndrome virus; ORF open reading frame; qPCR quantitative PCR.

Next, the residue(s) within GBP1 that could be cleaved by nsp4 was investigated. Previous studies have shown that equine arteritis virus (EAV) nsp4 preferentially cleaves the glutamic acid-glycine site in both the viral polyproteins and cellular target protein, but may also recognize the glutamic acid-serine or glutamic acid-alanine sites [47]. Next, the unique GBP1 cleavage site was identified. Based on the ~30 kDa molecular weight of the cleavage production and the high preference of nsp4 for E residues at the P1 position (Figure 9A) [48], cleavage sites may exist between amino acids 330 and 370. To confirm this hypothesis, a series of mutants with site-directed mutations within GBP1 at residues that might serve as nsp4 cleavage sites (E333, E338, E345 and E349) were constructed. The Flag-GBP1-wt and these mutant plasmids were co-transfected into HEK293T cells with HA-nsp4. As illustrated in Figure 9B, Flag-GBP1 was cleaved when nsp4 was co-expressed, producing a ~30 kDa cleavage protein. Meanwhile, the cleaved products disappeared following co-transfection of the Flag-GBP1-E338A mutation with HA-nsp4, while other mutants were still cleaved in the presence of HA-nsp4 (Figure 9B). A stable cell line expressing Flag-GBP1-E338A was established using the lentiviral expression system as described above. The mutant cell line was infected with 0.1MOI of PRRSV. However, PRRSV infection had no obvious cleavage effect of GBP1-E338A (Figure 9C). These results indicated that the E338-S350 pair of GBP1 is a unique cleavage site targeted by PRRSV nsp4.

### PRRSV nsp4 antagonizes the antiviral activity of GBP1 and nsp4-mediated cleavage of GBP1 impairs its antiviral activity

As PRRSV nsp4 was found to cleave GBP1 at the E338 site, the antiviral effects of GBP1 with the cleavage site mutation were evaluated. The Marc-145-GBP1-E338A-flag, Marc-145-GBP1_1-338_-flag and Marc-145-GBP1_339-591_-flag cell lines were used to determine the effect of GBP1 mutation on PRRSV infection. As shown in Figures 9D–F, the expression of PRRSV ORF7 mRNA, N protein and supernatant virus copies were significantly lower in the GBP1-E338A mutant group than the GBP1-wt group. In addition, the GBP1_1–338_ mutant maintained partial inhibitory ability against PRRSV, whereas the GBP1_339–591_ mutant completely abolished anti-PRRSV activity (Figures 9D–F), suggesting that PRRSV nsp4-mediated cleavage impairs the antiviral activity of GBP1.

Nsp4 is an important factor for PRRSV to antagonize the host immune response. Nsp4 interacts with several host cell proteins and consequently inhibits their antiviral activity [13, 20]. Since PRRSV nsp4 interacts with GBP1 and cleaves GBP1 to reduce its expression (Figures 6 and 8), whether nsp4 interfered with the anti-PRRSV activity of GBP1 was further investigated. Marc-145-Vector, Marc-145-GBP1-flag or Marc-145-GBP1-E338A-flag cells were transfected with or without plasmids expressing nsp4 for 24 h before infection with 0.1 MOI of PRRSV and incubation for another 36 h. qPCR results showed that the overexpression of Flag-GBP1 considerably reduced viral ORF7 mRNA, N protein expression and supernatant virus copies compared with the control group cells, while co-transfection of nsp4 reversed the antiviral activity of GBP1 as well as GBP1-E338A, as shown by the ORF7 mRNA, N protein expression and supernatant virus copies detection results (Figure 9G–I).

## Discussion

**Figure 10:**
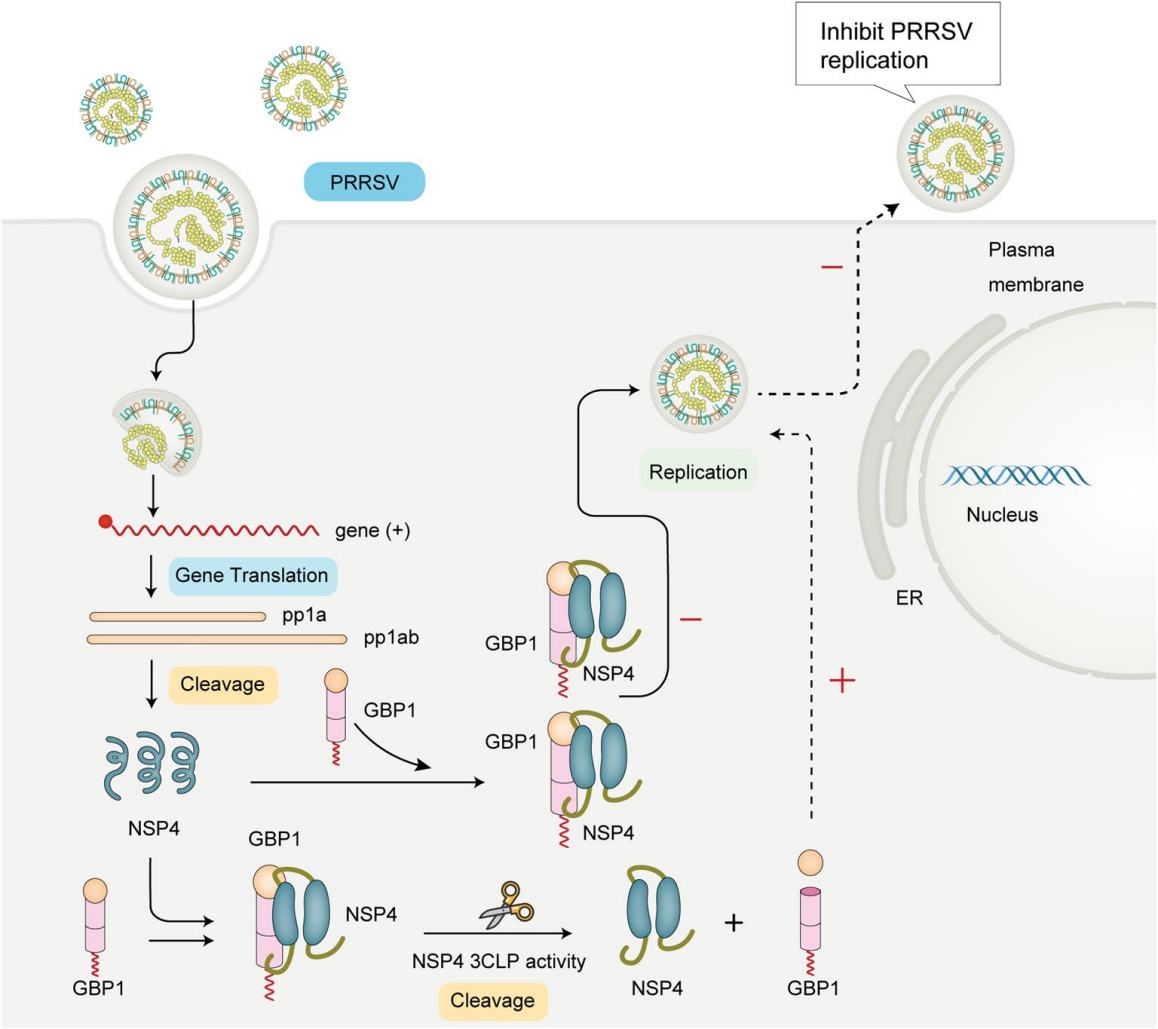
**Models of GBP1-mediated restriction of PRRSV replication.**

Persistent viral replication in host cells is governed by the cellular antiviral system and by the ability of the virus to evade antiviral responses. IFN-I are a family of cytokines that represent one of the first lines of defense against viral propagation and spread [49]. As a pathogen with significant negative implications to the porcine industry, HP-PRRSV has evolved complicated mechanisms to modulate and hijack host immune responses to facilitate self-replication [49, 50]. However, the role and molecular mechanism of most ISGs in the process of viral infection have not yet been clearly characterized. The present study demonstrated that porcine GBP1 is an effective anti-PRRSV host factor, and overexpression of GBP1 significantly inhibited PRRSV infection, while knockdown of endogenous GBP1 promoted viral infection. A mutation in the GTPase-catalyzing domain completely abolished antiviral activity against PRRSV, indicating that the antiviral activity of GBP1 was dependent on its GTPase activity (Figure 4). Further mechanistic analysis indicated that PRRSV nsp4 protein interacted with GBP1, which involved the GBP1 N-terminal domain and the nsp4 extra C-terminal α/β domain. Of note, overexpression of wild-type nsp4 lowers the antiviral action of GBP1 on PRRSV, and GBP1 was directly cleaved after PRRSV infection, revealing a novel strategy employed by PRRSV to attenuate the antiviral activity of ISGs (Figure 10).

The GBPs are a group of IFN-induced proteins, which belong to the GTPase family and are necessary for host immune response against numerous exogenous pathogens, including chlamydiae, toxoplasmas, bacteria and various viruses [51, 52]. Studies have shown that the GTPase domain of GBP1 is critical for antiviral effects. For instance, overexpression of GBP1 significantly suppresses HCV or IAV replication through its GTPase activity [28, 30]. In accordance with a previous study, our results suggested that the anti-PRRSV activity of GBP1 largely relied on its GTPase activity, since mutation of the K51 site, especially the R48 site, markedly attenuated the antiviral effect of GBP1 (Figure 4). This phenomenon can be viewed as one of the mechanisms by which GBP1 exerts its antiviral activity against PRRSV. It has been shown that the ΔCAAX mutant of GBP1 is not farnesylated and does not associate with the cell plasma membrane [39]. According to our results, deletion of the ΔCAAX motif of GBP1 still possessed anti-PRRSV effects (Figure 4), which was not consistent with a previous report that the ΔCAAX motif is not sufficient for antiviral effects during Kaposi’s sarcoma-associated herpesvirus infection [53], implying that for different pathogens, the molecular mechanisms by which GBP1 exerts its biological functions differ. Due to high expression of NSP4 and NSP7 might have an impact on proteins interaction, co-IP verification was performed to exclude the possibility of non-specificity interaction. As expected, only NSP4, but not NSP7, specifically interacted with GBP1 (Figure 6). GBP-1 is demonstrated to bind strongly to actin and is sufficient for actin cytoskeleton remodeling [39]. The present study found that GBP1 inhibited PRRSV infection and intercellular spread, which could be partially attributed to the disruption of the formation of actin filaments, emphasizing the intermediary role of actin in mediating the anti-PRRSV function of GBP1.

Similar to other RNA viruses in the order Nidovirales, such as EAV and severe acute respiratory syndrome coronavirus, PRRSV-encoded proteases play a prominent role in virus replication [46, 48]. PRRSV nsp4 is a 3CLSP encoded by PRRSV, which not only processes pp1a/pp1ab of the virus into 10 mature nsps, but also plays an important role in moderating host innate immune responses [54]. PRRSV nsp4 was reported to antagonize type I IFN production by cleaving adaptor IFN-κB promoter stimulator 1 and kinase NF-κB essential modulator [14]. PRRSV nsp4 has been found to cleave ZAP or DCP1 to antagonize their anti-PRRSV activity [20, 21]. The present study showed that nsp4 could directly cleave porcine GBP1 to impair its antiviral activity. Nevertheless, when the protease activity was deactivated, the degradation of GBP1 catalyzed by nsp4 was abolished, suggesting that the protease activity of nsp4 is crucial to antagonize the anti-PRRSV activity of GBP1. Previous studies have highlighted the type I IFN signaling pathway as the target of nsp4 [13, 14], thus taking these previous findings with the findings of the present study together, we conclude that nsp4 targets the IFN pathway and cleaves the ISGs to dampen the host defense. However, what needs to be stated is that although endogenous cleavage products of GBP1 in PRRSV-infected PAMs or Marc-145 cells were not detected in the current study, degradation of the GBP1 protein was observed in GBP1-overexpressing cells following viral infection. Thus, we speculate that the endogenous GBP1 cleavage products may be short-lived during PRRSV infection, or the cleavage products may be too weak to identify.

In mammalian cells, protein degradation primarily depends on the ubiquitin proteasome, apoptotic and autophagy-lysosome pathways [55, 56]. Thus, to verify whether the aforementioned three pathways participate in PRRSV infection-induced GBP1 degradation, specific inhibitors were used in the present study. PRRSV infection-induced GBP1 degradation was found to be independent of the ubiquitin proteasome, apoptotic and autophagy-lysosome pathways, suggesting that nsp4 protease activity specifically degrades GBP1. Mutations at H39A, D64A or S118A, which completely abolished the 3CLP activity of nsp4, and notably blocked its cleavage activity on GBP1, implying that 3CLP activity was critical for nsp4 to cleave GBP1. Previous studies showed that a preference for substrate cleavage by PRRSV Nsp4 is a Glu (E) residue at the P1 position [13]. Mutation of the cleavage site of nsp4 on GBP1 abrogated GBP1 cleavage and simultaneously augmented its antiviral activity, confirming the cleavage site of nsp4 on GBP1 in the current study. In fact, a large number of related studies have been carried out on nsp4 of different viruses, such as some human-hosted viruses-MERS [57], COVID-2019 [58], and some animal-hosted viruses-PEDV [59] and PoRV [60], nsp4 play different roles in viral replication and pathogenicity of different viruses. Researchers are already trying to develop antiviral drugs targeting nsp4 of different viruses, which may provide effective tools for the preventive, control and treatment of these viral diseases. PRRSV nsp4 cleaves GBP1 through its 3CLP activity to evade host antiviral immune reaction implying that the development of new drugs that can inhibit the enzymatic activity of nsp4 may help to enhance the antiviral ability of host cells or pigs will bring new hope for PRRS treatment.

The present study emphasizes the importance of PRRSV nsp4 3CLP activity in abrogating host antiviral immune reaction, and simultaneously suggests that nsp4 is an ideal target protein for the development of anti-PRRSV drugs. These studies provide a good theoretical basis for the research and development of antiviral drugs and suggest a bright prospect of drug development, although no effective drug targeting PRRSV nsp4 has been reported yet. Of note, as a non-structural protein of PRRSV, nsp4 plays an important role in the successful replication of PRRSV, however, it is not one of the final protein components of PRRSV virions, and this may lead to the fact that drugs targeting the enzymatic activity of nsp4 cannot completely block viral replication, which is also a limitation that must be considered when developing corresponding drugs.

In summary, our study revealed that GBP1 played an important role in suppressing PRRSV replication, while PRRSV nsp4 impaired the antiviral activity of GBP1 by cleaving it via nsp4’s 3CLP activity, thus developing a novel drug that inhibits PRRSV nsp4 3CLP activity may be viewed as an effective pathway for anti-PRRSV therapy.
